# YBX1-driven TUBB6 upregulation facilitates ocular angiogenesis via WNT3A-FZD8 pathway

**DOI:** 10.7150/thno.104573

**Published:** 2025-01-27

**Authors:** Ye-Ran Zhang, Wei-Qi Li, Zhong-Hong Zhang, Ru-Xu Sun, Hong-Jing Zhu, Hui-Ming Qian, Song-Tao Yuan, Yu-Liang Wang

**Affiliations:** 1 Department of Ophthalmology, The First Affiliated Hospital of Nanjing Medical University, Nanjing Medical University, Nanjing, China.; 2 Department of Ophthalmology, Zhongda Hospital, Southeast University, Nanjing, China.; 3 Department of Ophthalmology, Children's Hospital of Nanjing Medical University, Nanjing Medical University, Nanjing, China.; 4 The First Affiliated Hospital of Zhejiang University, Hangzhou, China.

**Keywords:** Pathological ocular angiogenesis, Endothelial cell, Single-cell RNA sequencing, Angiogenesis, Tip cell

## Abstract

**Background:** Pathological ocular neovascularization, a characteristic feature of proliferative ocular diseases, is a primary contributor to global vision impairment. The dynamics of tubulin are crucial in maintaining ocular homeostasis, closely linked to cellular proliferation and angiogenesis. Elucidating the molecular mechanisms driving this process is vital for formulating effective therapeutic strategies.

**Methods:** Multiple transcriptome analyses revealed upregulation of endothelial tubulin beta-6 chain (*Tubb6*) in oxygen-induced retinopathy (OIR) and laser-induced choroidal neovascularization (CNV) mice models. Transwell migration assay, wound healing assay, tube formation assay, flow cytometry, and immunofluorescent staining were employed to identify the role of TUBB6 knockout (KO) *in vitro*. The effects of Tubb6 silencing on retinal angiogenesis and choroidal neovascularization were subsequently evaluated.

**Results:** We identified upregulated *Tubb6* expression in retinas from OIR mice through combination analyses of single-cell RNA sequencing (scRNA-Seq) and bulk RNA-Seq. The RNA expression profiles of endothelial cells (ECs) from proliferative diabetic retinopathy (PDR) patients and neovascular age-related macular degeneration (nAMD) patients also exhibited an elevation in *TUBB6*. Notably, Tubb6 was abundantly expressed in ECs and pericytes, and was predominantly localized to proliferative ECs and vascular tip cells. Functional studies demonstrated that TUBB6 knockdown reduced the expression of proliferative and tip cell markers *in vitro.* Tubb6 deficiency decreased vascular sprouting and tip cell formation of OIR mice retina and retarded CNV progression *in vivo*. Mechanistically, YBX1, an RNA-binding protein, was identified as an upstream regulator of TUBB6 via binding to its 3' untranslated region (3'UTR) and maintaining mRNA stability. Transcriptome analysis further linked TUBB6 to the activity of WNT pathway. TUBB6 silencing suppressed the WNT signaling pathway, with WNT3A and FZD8 identified as downstream targets.

**Conclusions:** Collectively, our research shed light on the pivotal function of TUBB6 in maintaining ocular homeostasis and uncovered the YBX1-TUBB6-WNT3A/FZD8 pathway's involvement in sprouting angiogenesis. Targeting TUBB6 and developing its specific inhibitor could pioneer new approaches for treating ocular microvascular diseases.

## Introduction

Angiogenesis is a crucial physiological process involved in maintaining homeostasis. With the advancements in single-cell RNA sequencing (scRNA-Seq) technology, endothelial cells (ECs) demonstrate significant heterogeneity and can be classified into various subtypes, including tip cells, proliferative ECs, immature ECs, mature ECs, transitioning ECs, as well as arterial and venous ECs 1, 2. Tip cells are specialized endothelial cells located at the extremity of newly formed capillaries. They extend filopodia and guide the growth of capillaries, playing a pivotal role in tissue vascularization, which makes them a primary target for angiogenic therapies 3. Vascular endothelial growth factor (VEGF) and other stimulants can activate endothelial cells, prompting them to differentiate into tip cells, which subsequently secrete proteases to degrade the basement membrane 3, 4. During the process of vessel sprouting, the navigating tip ECs lead the way, while proliferating ECs proliferate and migrate, elongating vessel sprouts 2, 5, 6. Pathological angiogenesis has been associated with the development of several human diseases, such as cerebral small vessel disease, Alzheimer's disease, and ocular disorders. The mechanisms of EC activation and angiogenesis are still largely unknown, serving as the focus of our research.

Ocular microvascular diseases are primary causes of severe vision loss in children and adults worldwide, featured by aberrantly proliferating vascular tuft structure 7, 8. VEGF plays a crucial role in the pathogenesis of ocular angiogenesis, rendering it an essential therapeutic target 9-11. The introduction of anti-VEGF agents has significantly altered the prognosis of ocular angiogenesis diseases 12, 13. Despite anti-VEGF therapy becoming the standard treatment for pathological ocular neovascularization, a considerable proportion of patients do not respond adequately to this therapy 14, 15. Additionally, the necessity for frequent administration of anti-VEGF agents results in a gradual reduction in their efficacy over time 12, 16, 17. These clinical observations implicate that additional critical factors contribute to the promotion of ocular angiogenesis. Consequently, identifying new targets implicated in the pathogenesis of ocular neovascularization is essential for the advancements of future therapies. Recent advancements in single-cell RNA sequencing (scRNA-Seq) have enabled detailed analysis of cellular and molecular mechanisms within specific tissues 18-20.

Microtubules are fundamental elements of the eukaryotic cytoskeleton, essential for cell division, morphology, motility, and angiogenesis 21, 22. Despite their diverse functions, microtubules exhibit a highly conserved structure composed of nearly identical molecular subunits: the tubulin proteins. The properties and functions of the microtubule cytoskeleton are governed by various tubulin isotypes and a range of post-translational modifications, collectively termed the 'tubulin code' 23. The tubulin dynamics plays a pivotal role in ocular homeostasis and Tubb3 (beta-III tubulin) has been identified as a marker for assessing structural integrity of retinal ganglion cells (RGCs) in the retina 24, 25. Kobayashi *et al.* reported that detyrosination of α-tubulin in ECs significantly suppressed angiogenesis 22. Retinal stem cells (RSCs) co-cultured with oxygen-induced retinopathy of prematurity (OIR-ROP) retinal tissues could be induced to differentiate into cells expressing β-tubulin and protein kinase C (PKC) and promote the expression of angiogenin-1 (Ang-1) and insulin-like growth factor-1 (IGF-1), thus enhancing proliferation and angiogenesis 26.

Based on integrated analysis of single-cell RNA sequencing and bulk transcriptomics, we found that the expression of *Tubb6*, encoding the tubulin beta-6 chain, was significantly upregulated in vascular ECs in both the OIR and the CNV mouse models. Notably, TUBB6 was predominantly localized to proliferative ECs and vascular tip cells. This expression pattern suggests a critical role of TUBB6 in regulating angiogenesis, which was also observed in the central nervous system (CNS) vascular endothelium. *In vitro* experiments, TUBB6 knockout (KO) impeded VEGF-induced endothelial proliferation, migration, and tube formation. *In vivo*, TUBB6 knockdown decreased neovascular and avascular areas in OIR retina and reduced CNV progression. YBX1 was found to positively regulate TUBB6 expression via binding to its 3' untranslated region (3'UTR). Mechanistically, TUBB6 regulated ocular angiogenesis through the activation of WNT3A/FZD8 pathway. Our study is crucial for a deeper understanding of the molecular networks involved in vascular sprouting and highlights the promise of the YBX1-TUBB6-WNT3A/FZD8 axis as a therapeutic target for pathological ocular angiogenesis.

## Results

### TUBB6 expression is upregulated in ECs from clinical PDR patients and OIR mice, predominantly localized in vascular cells

**Figure 1:**
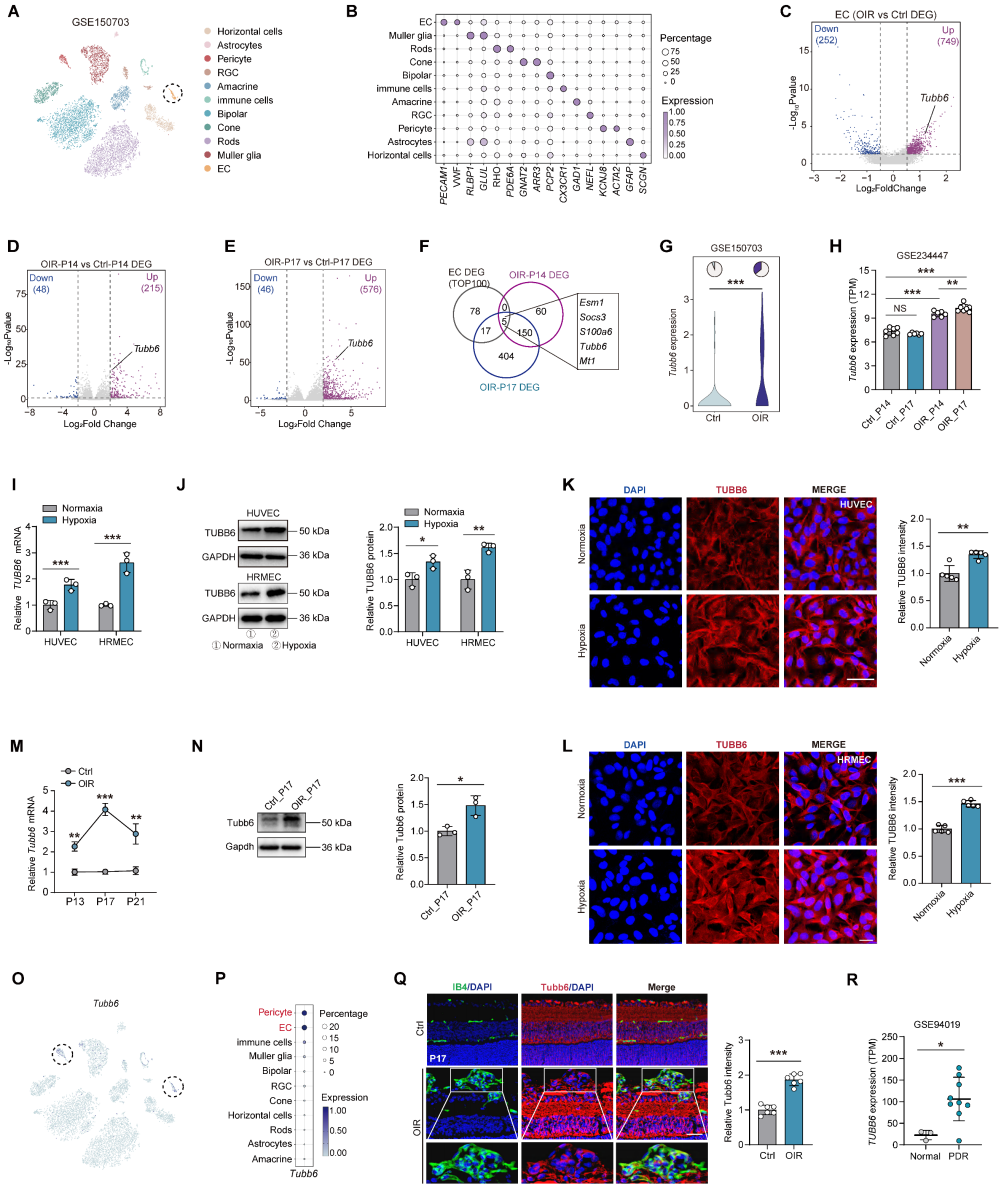
** TUBB6 expression is upregulated in ECs from clinical PDR patients and OIR mice, predominantly localized in vascular cells. (A)** tSNE plot showing retinal cell types from control and OIR retinas (GEO accession: GSE150703). **(B)** Dot plot of key markers used to identify cell types in the tSNE plot. **(C)** Volcano plots showing DEGs of OIR ECs vs control ECs from **Figure 1A**. **(D-E)** Volcano plots showing DEGs of OIR vs control retinas (GEO accession: GSE234447) at P14 **(D)** and P17 **(E)** respectively. **(F)** Venn diagram of upregulated DEGs from **C-E**. **(G)** Visualization of *Tubb6* expression in the ECs from **Figure 1A**. The pie charts showing the percentage of *Tubb6*^+^ (counts > 0) cells and the violin plot showing relative *Tubb6* expression in ECs from the control and OIR groups. **(H)** Visualization of *Tubb6* expression in the OIR retinas from **Figure 1D-E** at P14 (*n* = 6) and P17 (*n =* 8) compared to those from normal subjects at P14 (*n* = 7) and P17 (*n =* 6) (GEO accession: GSE234447). **(I-J)** mRNA **(I)** and protein **(J)** levels of TUBB6 in the hypoxia-treated HUVECs and HRMECs compared with their respective control groups. *n* = 3 per group. **(K-L)** Immunofluorescence staining of TUBB6 in the hypoxia-treated HUVECs **(K)** and HRMECs **(L)**. *n* = 5 per group. Scale bar: 50 μm **(K)** and 20 μm** (L)**. **(M-N)** mRNA **(M)** and protein **(N)** levels of Tubb6 in the OIR retinas and normal controls. *n* = 3 per group. **(O-P)** Feature plot **(O)** and dot plot **(P)** showing *Tubb6* expression across distinct retinal cell types. **(Q)** Immunofluorescence staining of Tubb6 and IB4 in retinal cryosections of OIR mice and normal controls. *n* = 6 per group. Scale bar: 50 μm. **(R)** Relative *TUBB6* expression in retinal microvascular ECs from patients with PDR (*n* = 9) compared with those from normal subjects (*n* = 3) (GEO accession: GSE94019). 'TPM' stands for 'Transcripts per kilobase million'. Data represent different numbers (*n*) of biological replicates. Data are shown as mean ± SD. Two-tailed Student's T test is used in **I-L** and **R**. *p < 0.05; **p < 0.01; and ***p < 0.001.

To elucidate the adaptive responses and structural alterations of retinal vessels during pathological angiogenesis, we re-analyzed the publicly accessible OIR mouse single-cell dataset (GEO accession: GSE150703) 27. Retinal cell types were visualized using t-distributed stochastic neighbor embedding (t-SNE) plot and identified by classic markers **(Figure 1A-B)**. Endothelial cells (ECs) were extracted from the single-cell object for differential gene expression analysis **(Figure 1C)**. We also compared the differentially expressed genes (DEGs) of OIR mouse retina versus the control at P14 and P17 (GEO accession: GSE234447) **(Figure 1D-E)** 28. By intersecting up-regulated genes from 3 comparisons **(Figure 1F)**, we found that 5 shared genes and *Tubb6* emerged as a potential target based on previous studies **(Figure 1F-H)**. *In vitro*, qPCRs, western blots, and immunofluorescent staining revealed that TUBB6 expression was markedly increased in hypoxia-treated human umbilical vein endothelial cells (HUVECs) and human retinal microvascular endothelial cells (HRMECs) compared to normoxia controls** (Figure 1I-L)**. Moreover, qPCRs and western blots revealed that TUBB6 expression was notably increased in retinas of OIR mice, reaching its top at P17** (Figure 1M-N)**. We further investigated the distribution of TUBB6 across retinal cell types. Single-cell data showed that *Tubb6* was mainly expressed in vascular ECs and pericytes, both of which are vital cellular components of vasculature **(Figure 1O-P)**. In retinal cryosections, immunofluorescence staining also confirmed the upregulation of Tubb6 **(Figure 1Q)**. Bioinformatics analysis of publicly available transcriptomic datasets (GEO accession: GSE94019, 29) demonstrated a notable elevation of *TUBB6* expression in retinal microvascular ECs from patients with proliferative diabetic retinopathy (PDR) in contrast to normal subjects **(Figure 1R)**. Overall, TUBB6 was upregulated in ECs of clinical PDR patients and OIR retina, indicating the pivotal role of TUBB6 in the pathogenesis of retinal neovascularization.

### TUBB6 expression is elevated in ECs of clinical nAMD patients and laser-induced CNV models

**Figure 2:**
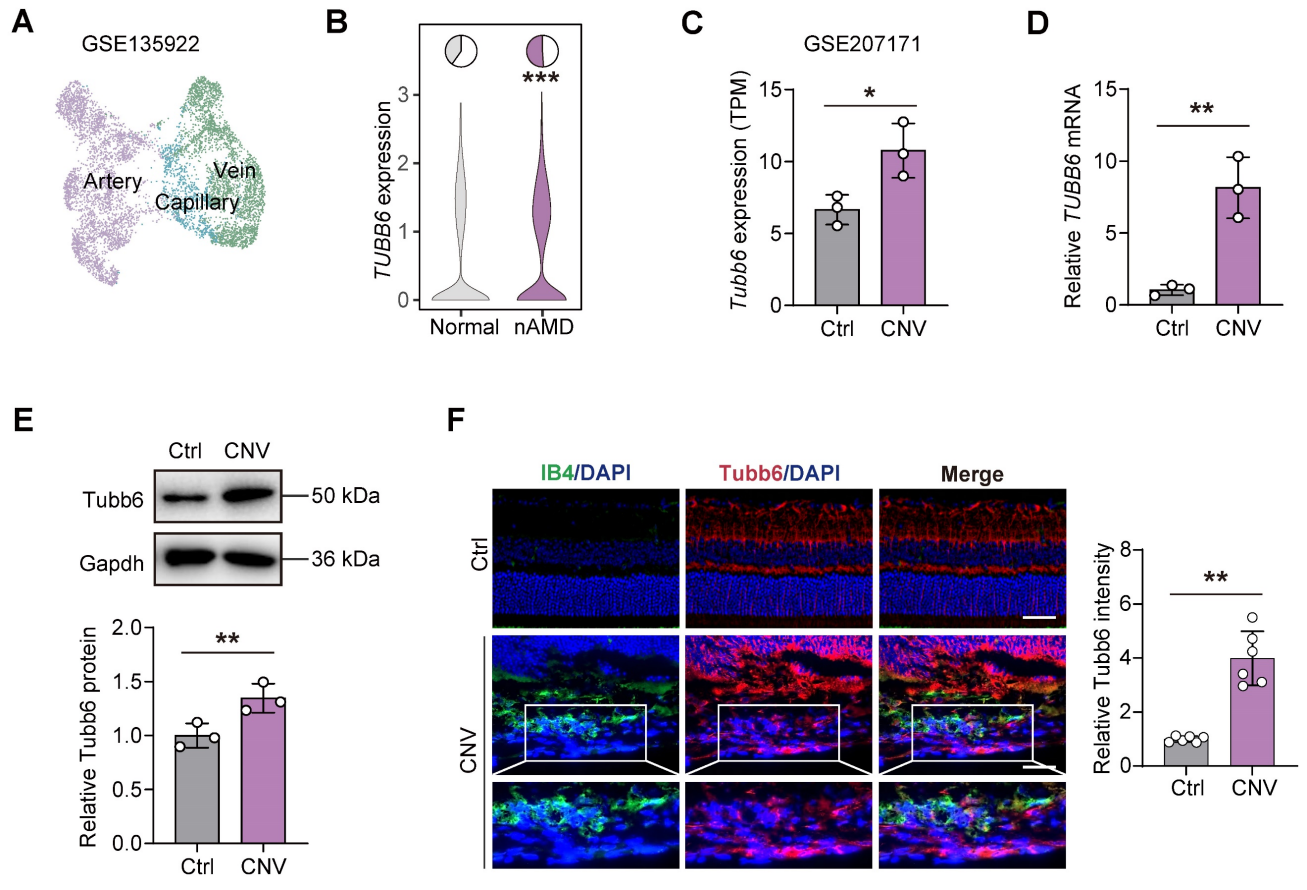
** TUBB6 expression is elevated in ECs of clinical nAMD patients and laser-induced CNV models. (A)** UMAP plot showing ECs from publicly CD31-enriched scRNA-Seq data of nAMD patients (GEO accession: GSE135922). **(B)** Expression of *TUBB6* in ECs from nAMD patients and normal subjects. The pie charts showing the percentage of *TUBB6*^+^ cells (counts > 0) and the violin plot showing relative *TUBB6* expression in nAMD patients compared to the controls. **(C)** Relative expression of *Tubb6* in the laser-induced CNV mice compared to age-matched controls (GEO accession: GSE207171). 'TPM' stands for 'Transcripts per kilobase million'. *n* = 3 per group. **(D-E)** mRNA **(D)** and protein **(E)** levels of Tubb6 in CNV choroids. *n* = 3 per group. **(F)** Immunofluorescence staining of Tubb6 and IB4 in CNV choroids. *n* = 6 per group. Scale bar: 50 μm (control) and 20 μm (OIR). Data represent different numbers (*n*) of biological replicates. Data are shown as mean ± SD. Two-tailed Student's T test is used in **C-F**. *p < 0.05; **p < 0.01.

Choroid neovascularization (CNV) and the resulting ocular angiogenesis are pathological hallmarks of neovascular age-related macular degeneration (nAMD) 30. To explore the role of TUBB6 in the choroid, publicly available single-cell RNA sequencing data (GEO accession: GSE135922) of RPE-choroid complex from patients with nAMD and normal adults were re-analyzed **(Figure 2A)** 31. *TUBB6* expression was found to be upregulated in the ECs of nAMD patients** (Figure 2B)**. We also investigated the bulk RNA-Seq from mouse models of laser-induced CNV (GEO accession: GSE207171) and found that in accordance with the scRNA-Seq data of clinical samples, *Tubb6* was upregulated in laser-induced CNV model **(Figure 2C)** 30. qPCRs and western blots revealed that Tubb6 level was markedly increased in RPE-choroid complexes obtained from the CNV mice compared to the control group **(Figure 2D-E)**. Consistent with above findings, immunofluorescence of choroid cryosections showed that Tubb6 was upregulated in the CNV mice **(Figure 2F)**. Collectively, TUBB6 was up-regulated in ECs from clinical AMD samples and CNV models* in vivo*.

### TUBB6, localized to tip cells and proliferative ECs, governs tip cell formation and endothelial cell proliferation

**Figure 3:**
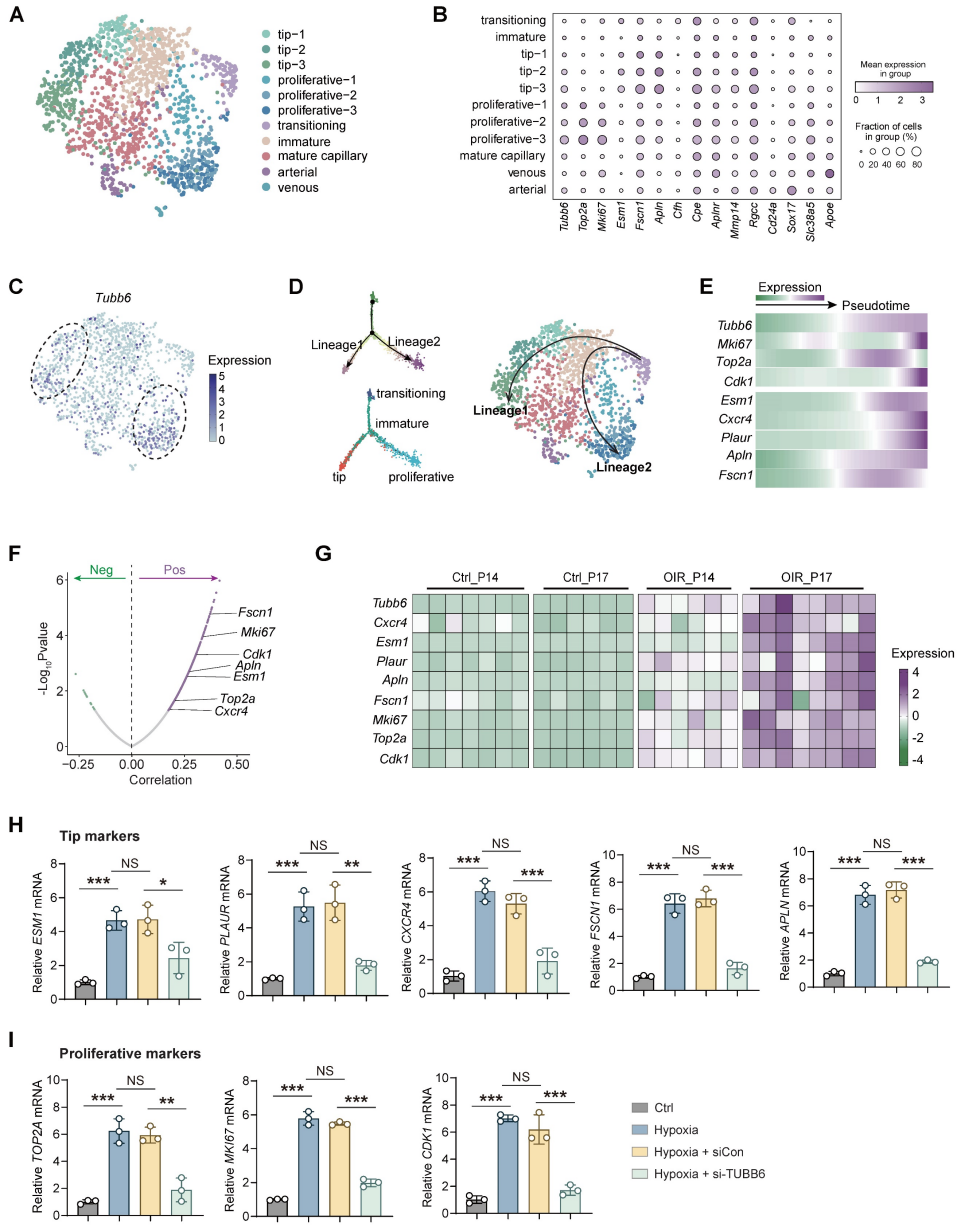
** TUBB6, localized to tip cells and proliferative ECs, governs tip cell formation and endothelial cell proliferation. (A)** UMAP plot showing distinct EC subsets in Cd31-enriched scRNA-Seq of OIR retina at P14 (GEO accession: GSE174400). **(B)** Dot plot of key markers used to identify distinct EC subsets. **(C)** Feature plot showing *Tubb6* expression across distinct EC subsets. **(D)** Pseudotime-ordered analyses of EC clusters from OIR retina. **(E)** Heatmap showing representative genes in lineage 1 and lineage 2 along with the pseudotime. **(F)** Pearson correlation analysis between *Tubb6* and proliferative markers and tip cell markers was conducted. 'Pos' stands for 'positive correlation', 'Neg' stands for 'negative correlation'. **(G)** Heatmap showing expression levels of *Tubb6* and interested genes in OIR retinas from GSE234447. **(H-I)** RNA expression of tip markers **(H)** and proliferative cell markers **(I)** in HUVECs with TUBB6 knockdown under hypoxic condition. *n* = 3 per group. Data represent different numbers (*n*) of biological replicates. Data are shown as mean ± SD. One-way ANOVA followed by Bonferroni's test is used in **H-I**. NS: not significant (p > 0.05); *p < 0.05; **p < 0.01; and ***p < 0.001.

Vascular endothelial cells play a pivotal role in pathological angiogenesis 32. Nonetheless, the transcriptomic and signaling heterogeneity involved in this process are not fully elucidated. Basic ECs could be further divided into functionally and molecularly distinct subtypes in single-cell RNA analysis 33. Given the specific vascular localization of Tubb6, Cd31-enriched scRNA-Seq data (GEO accession: GSE174400) of OIR mice retina was re-analyzed 34. Retinal ECs were further subdivided into different subpopulations, including tip cells, proliferative ECs, immature ECs, mature ECs, transitioning ECs, arterial ECs, and venous ECs** (Figure 3A-B)** according to previous studies 2. *Tubb6* was found to be highly expressed in tip cells and proliferative ECs compared to other subtypes** (Figure 3B-C)**. Single-cell trajectory analysis has revealed two distinct lineages in endothelial cell differentiation. During the transition from an immature state to either tip or proliferative states, apart from the up-regulation of classic tip and proliferative markers, the expression of *Tubb6* also increased along with pseudotime **(Figure 3D-E)**. Correlation analysis indicated that *Tubb6* was positively associated with classic proliferative (*Mki67, Top2a, Cdk1*) and tip markers (*Cxcr4, Esm1, Apln, Fscn1*) **(Figure 3F)**. Similarly, *Tubb6*, proliferative marker, as well as tip cell marker were up-regulated in OIR retina from **Figure 1D-E** along with time **(Figure 3G)**. To validate the *in vitro* function of TUBB6, we transfected HUVECs with TUBB6 small interfering RNA (si-TUBB6) or scramble siRNA (siCon). The TUBB6 knockdown efficiency was detected through qPCR and western blot, demonstrating an approximate knockdown efficiency of 80%** (Supplementary Figure S1A-B)**. qPCR assay revealed that in TUBB6-knockdown HUVECs, the expression of tip cell markers **(Figure 3H)** and proliferation markers **(Figure 3I)** was downregulated compared to the control group, corroborating the findings from single-cell RNA sequencing analysis.

We further examined the distribution of Tubb6 expression in the vasculature of the central nervous system (CNS). A single-cell expression dataset of ECs in the developing brain (GEO accession: GSE111839) from P7 mice was reanalyzed 33. Results revealed relatively high expression of *Tubb6* in tip cells and proliferative ECs compared to other EC subtypes **(Supplementary Figure S2A-C)** and its co-expression with tip **(Supplementary Figure S2D-H)** and proliferative markers **(Supplementary Figure S2I-K)**. This expression pattern is consistent with that observed in the OIR retina, indicating the critical role of Tubb6 in sprouting angiogenesis throughout the whole CNS.

### TUBB6 knockout impedes VEGF-induced angiogenesis in cultured ECs *in vitro*

**Figure 4:**
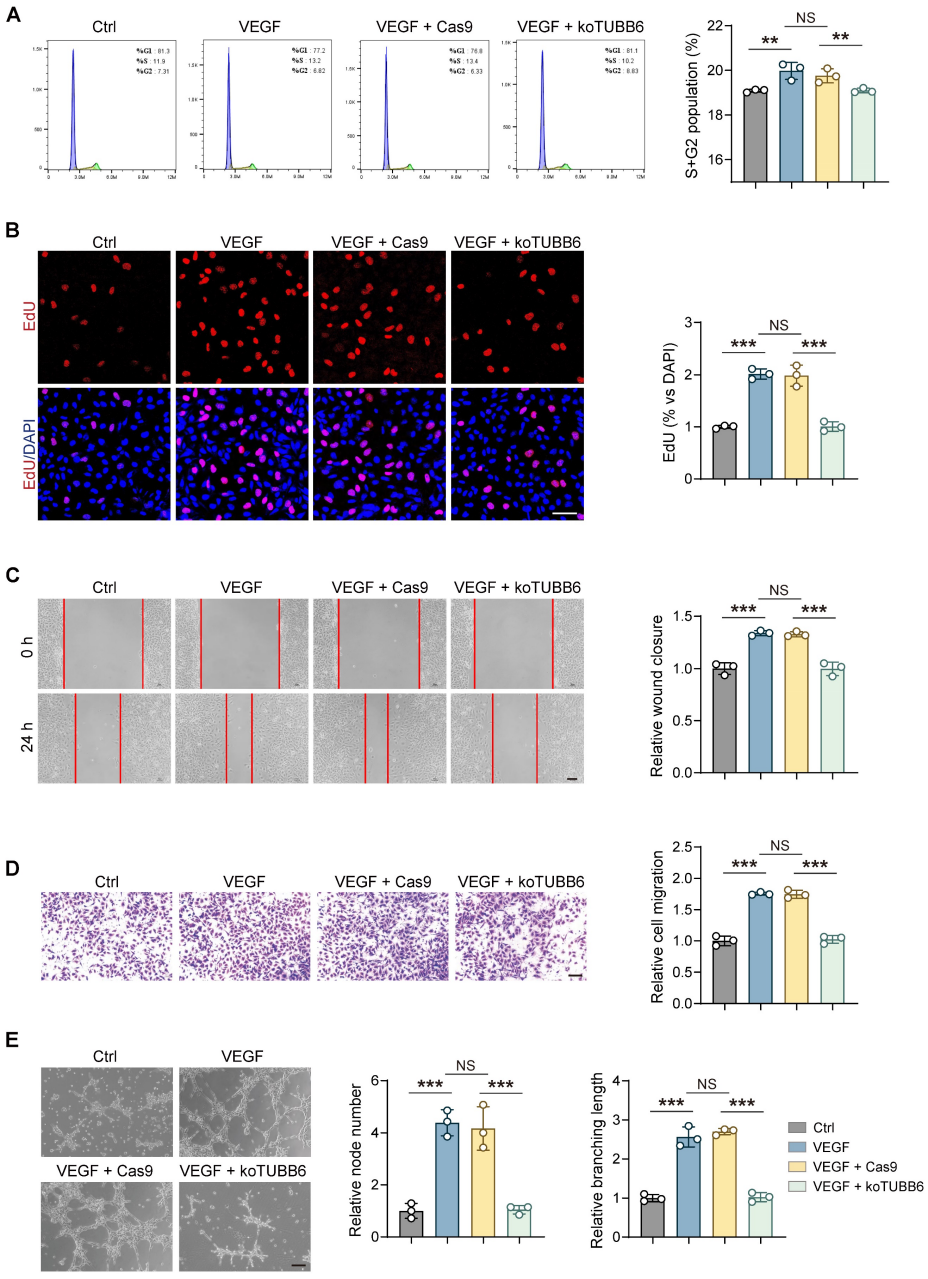
** TUBB6 knockout impedes VEGF-induced angiogenesis in cultured ECs *in vitro.* (A)** Flow cytometric analyses of cell cycle progression in control HUVECs and HUVECs treated with VEGF, VEGF plus Cas9, VEGF plus TUBB6-knockout. *n* = 3 per group. **(B)** Edu assays of HUVECs with distinct treatments. *n* = 3 per group. Scale bar: 50 μm. **(C)** Representative images of scratch-induced migration of HUVECs with distinct treatments. *n* = 3 per group. Scale bar: 50 μm. **(D)** Transwell assays of HUVECs with distinct treatments. *n* = 3 per group. Scale bar: 50 μm. **(E)** Representative tube formation images of HUVECs with distinct treatments. *n* = 3 per group. Scale bar: 50 μm. Data represent different numbers (*n*) of biological replicates. Data are shown as mean ± SD. One-way ANOVA followed by Bonferroni's test is used in **A-E**. NS: not significant (p > 0.05); **p < 0.01; and ***p < 0.001.

To further clarify the involvement of TUBB6 in proliferative retinopathy, the CRISPR/Cas9 gene editing method was employed to knockout (KO) TUBB6 in HUVECs. A small guide RNA (sgRNA) targeting the second exon of human TUBB6 gene (5′-AAGTTTTGGGAAGTGATCAG-3′) was designed by Genechem (Shanghai, China) and the related sgRNA sequence was supplied in **Supplementary Table S1**. We established stable TUBB6-knockout HUVECs (koTUBB6) and the control group (Cas9), and validated the knockout effect via western blotting **(Supplementary Figure S3)**. We utilized recombinant human VEGF165 as a stimulation of angiogenic phenotypes*.* Flow cytometric analyses confirmed that VEGF-induced cell cycle progression, demonstrated by percentage of cells in S and G2/M phases, was suppressed in TUBB6-knockout HUVECs **(Figure 4A)**. The proliferative capability was detected with 5-Ethynyl-2'-deoxynridine (EdU) assay. TUBB6-knockout treatment impeded VEGF-induced HUVEC proliferation *in vitro* and decreased the percentage of EdU-positive nuclei **(Figure 4B)**. Moreover, enhanced tube formation and migration induced by VEGF165 stimulation were suppressed in TUBB6 knockout HUVECs **(Figure 4C-E)**. We thus speculated that TUBB6 is associated with angiogenesis and TUBB6 silencing impedes *in vitro* angiogenic phenotypes.

### Tubb6 deficiency reduces pathological neovascularization and tip cell formation in OIR model

**Figure 5:**
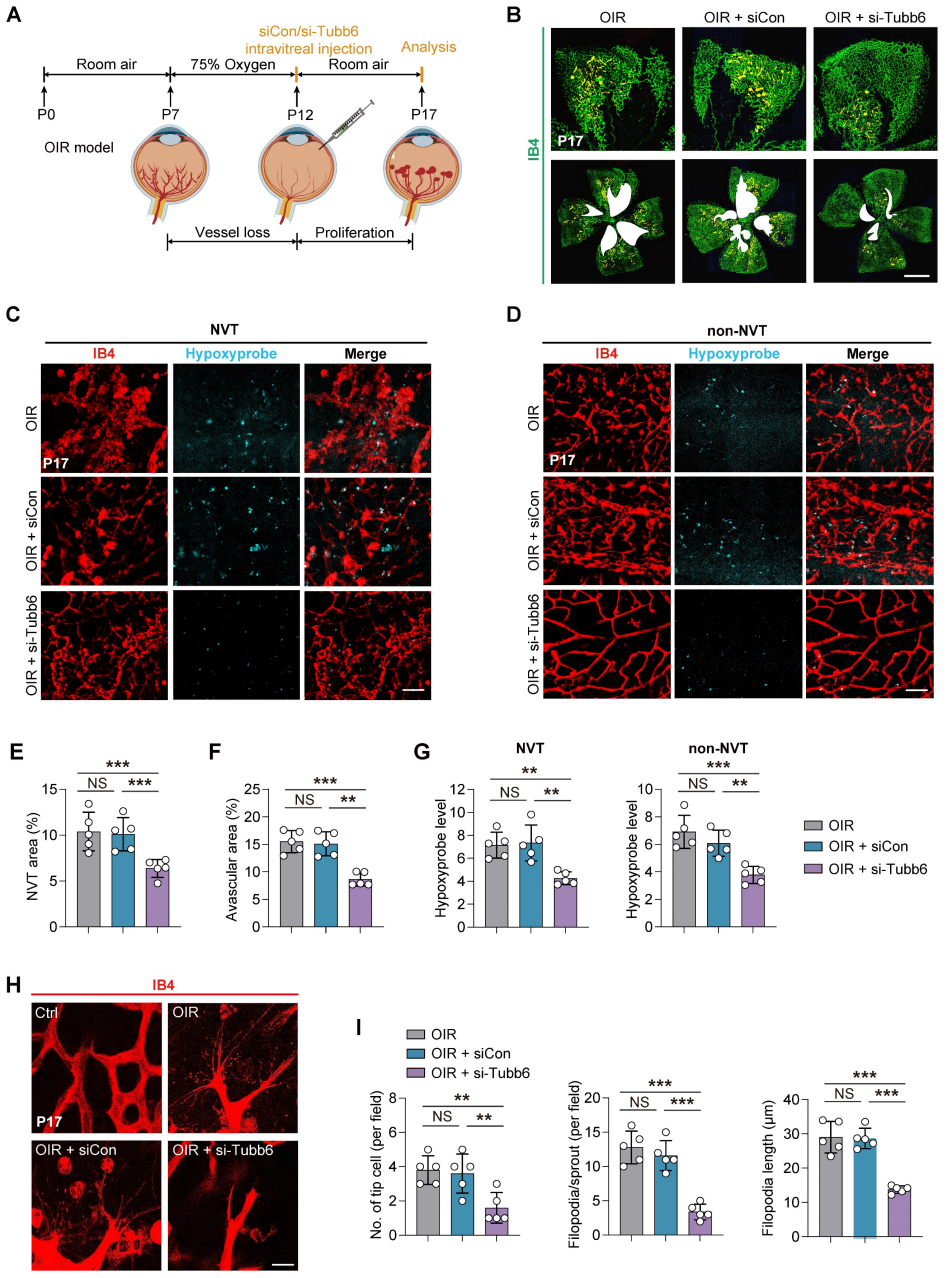
** Tubb6 deficiency reduces pathological neovascularization and tip cell formation in OIR model. (A)** Experimental scheme for **B-I**. **(B)** Fluorescent staining of IB4 in retinal flat mounts from OIR mice and OIR mice injected with siCon/si-Tubb6. *n* = 5 per group. Scale bar: 50 μm. **(C-D)** Fluorescent staining of hypoxyprobe in NVT area **(C)** and non-NVT **(D)** area from OIR mice and OIR mice injected with siCon/si-Tubb6. *n* = 5 per group. Scale bar: 50 μm. **(E-F)** Quantification results of NVT area **(E)** and avascular area **(F)** were shown. *n* = 5 per group. **(G)** Quantification results of hypoxyprobe fluorescence intensity in NVT area (left) and non-NVT area (right) were shown. *n* = 5 per group. **(H-I)** High-resolution images of IB4-stained retinas showing tip cells from OIR mice and OIR mice injected with siCon/si-Tubb6. *n* = 5 per group. Scale bar: 50 μm. Data represent different numbers (*n*) of biological replicates. Data are shown as mean ± SD. One-way ANOVA followed by Bonferroni's test is used in **B-I**. NS: not significant (p > 0.05); **p < 0.01; and ***p < 0.001.

To further investigate the role of Tubb6 in pathological angiogenesis, we initially utilized the oxygen-induced retinopathy (OIR) model 35, 36. Intravitreal injections of siCon/si-Tubb6 were administered to mice at postnatal day 12 (P12, the beginning of pro-angiogenic stage), and retinas were collected at postnatal day 17 (P17), prior to the regression of pathological vessels **(Figure 5A)**. The silencing efficiency of Tubb6-siRNA in mice was confirmed via qPCR and western blot **(Supplementary Figure S1E-F)**. Additionally, TUBB6 sequence was highly conserved between human and mice **(Supplementary Figure S4)**. OIR mice with Tubb6 knockdown demonstrated a marked decrease in neovascular tufts (NVTs) and avascular areas in retina as compared with their littermate controls **(Figure 5B, E-F)**. Hypoxia, as the central driver of retinal angiogenesis 37, 38, was detected using the hypoxyprobe, a hypoxia molecular probe for detecting hypoxia 39. A notable reduction in hypoxyprobe signaling was observed in both NVT and non-NVT regions following Tubb6 knockdown **(Figure 5C-D, G)**. This suggested Tubb6 knockdown mitigated retinal hypoxic conditions in mice retina. Isolectin B4 (IB4) staining of retinal flat mounts demonstrated Tubb6 inhibition led to decreased sprouting angiogenesis in OIR mice, evidenced by a reduction in the number of endothelial tip cells and shorter filopodia outgrowth **(Figure 5H-I)**, which is consistent with our *in vitro* findings. Collectively, our data suggested that Tubb6 knockdown *in vivo* alleviated pathological NVTs development and endothelial tip cell formation in the vessel proliferation period of OIR model.

### Tubb6 deficiency ameliorates retinal pericyte function and vascular integrity *in vivo*

**Figure 6:**
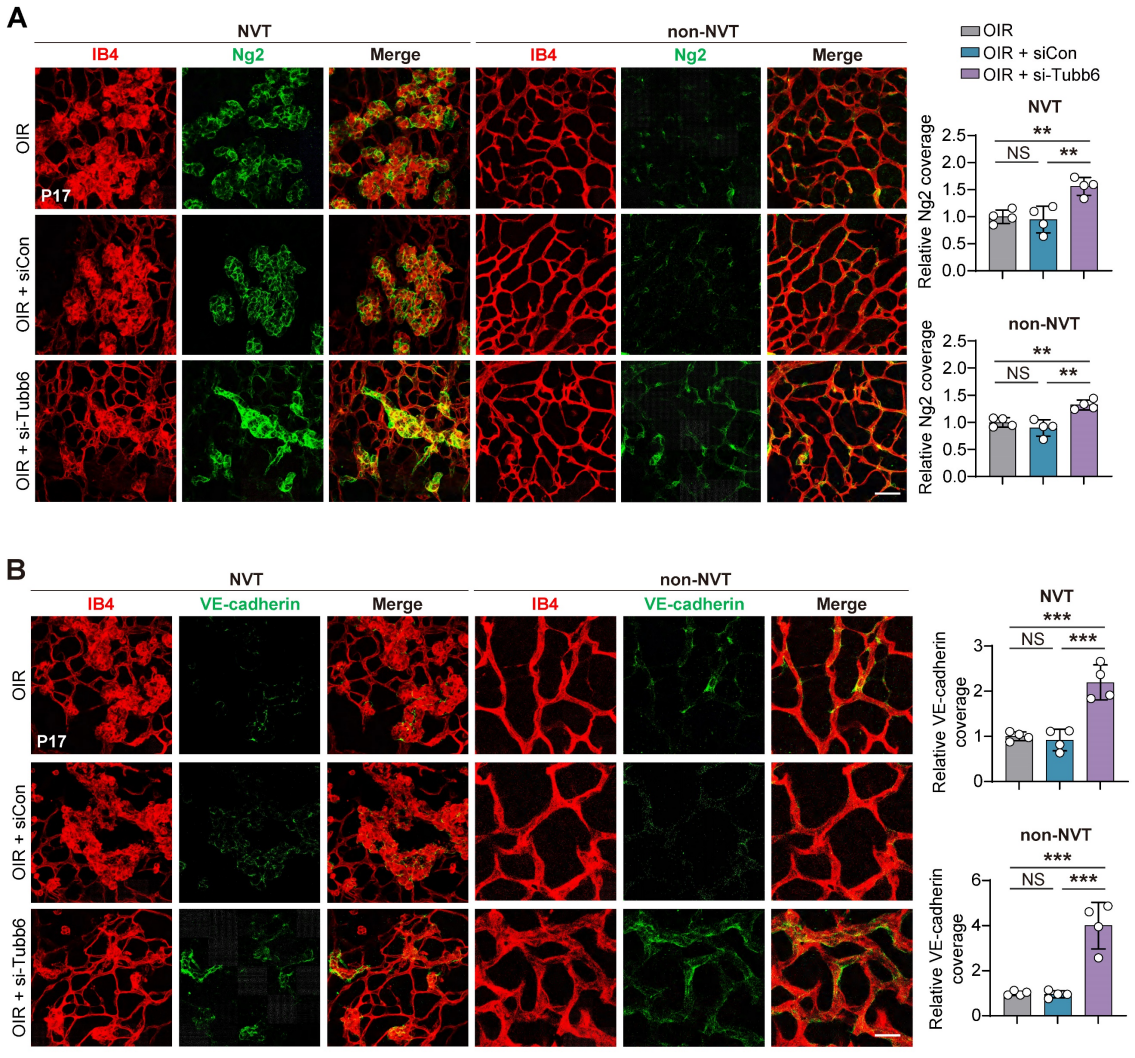
** Tubb6 deficiency ameliorates retinal pericyte function and vascular integrity *in vivo*. (A)** Fluorescent staining of IB4 and Ng2 in retinal flat mounts from OIR mice and OIR mice injected with siCon/si-Tubb6 in NVT area (left) and non-NVT (right) area at P17. *n* = 4 per group. Scale bar: 50 μm. **(B)** Fluorescent staining of IB4 and VE-cadherin in retinal flat mounts from OIR mice and OIR mice injected with siCon/si-Tubb6 in NVT area (left) and non-NVT (right) area at P17.* n* = 4 per group. Scale bar: 50 μm. Data represent different numbers (*n*) of biological replicates. Data are shown as mean ± SD. One-way ANOVA followed by Bonferroni's test is used in **A-B**. NS: not significant (p > 0.05); **p < 0.01; and ***p < 0.001.

Given that *Tubb6* expression was observed in both ECs and pericytes as shown in **Figure 1O-P**, we proceeded to examine the impact of Tubb6 on retinal pericyte functionality and vascular integrity *in viv*o. IB4 and neuron-glial antigen 2 (Ng2) immunofluorescence staining was conducted to assess the pericyte coverage in retinal vessels. Knockdown of Tubb6 in OIR retinas restored pericyte coverage compared to both the OIR and scramble groups, particularly in the NVT area **(Figure 6A)**. Retinal flat mounts were stained with vascular-endothelial-specific cadherin (VE-cadherin) to visualize endothelial adherens junctions, as VE-cadherin is critical for regulating cellular junctions and maintaining endothelial barrier integrity 40, 41. Enhanced VE-cadherin coverage was observed in both NVT and non-NVT vessels in retinal flat mounts from mice injected with si-Tubb6 in contrast to the OIR group **(Figure 6B)**. These data indicated that Tubb6 silencing restored vascular integrity disrupted by hypoxia treatment and pathological angiogenesis *in vivo*.

### Tubb6 deficiency inhibits vascular leakage and CNV progression *in vivo*

**Figure 7:**
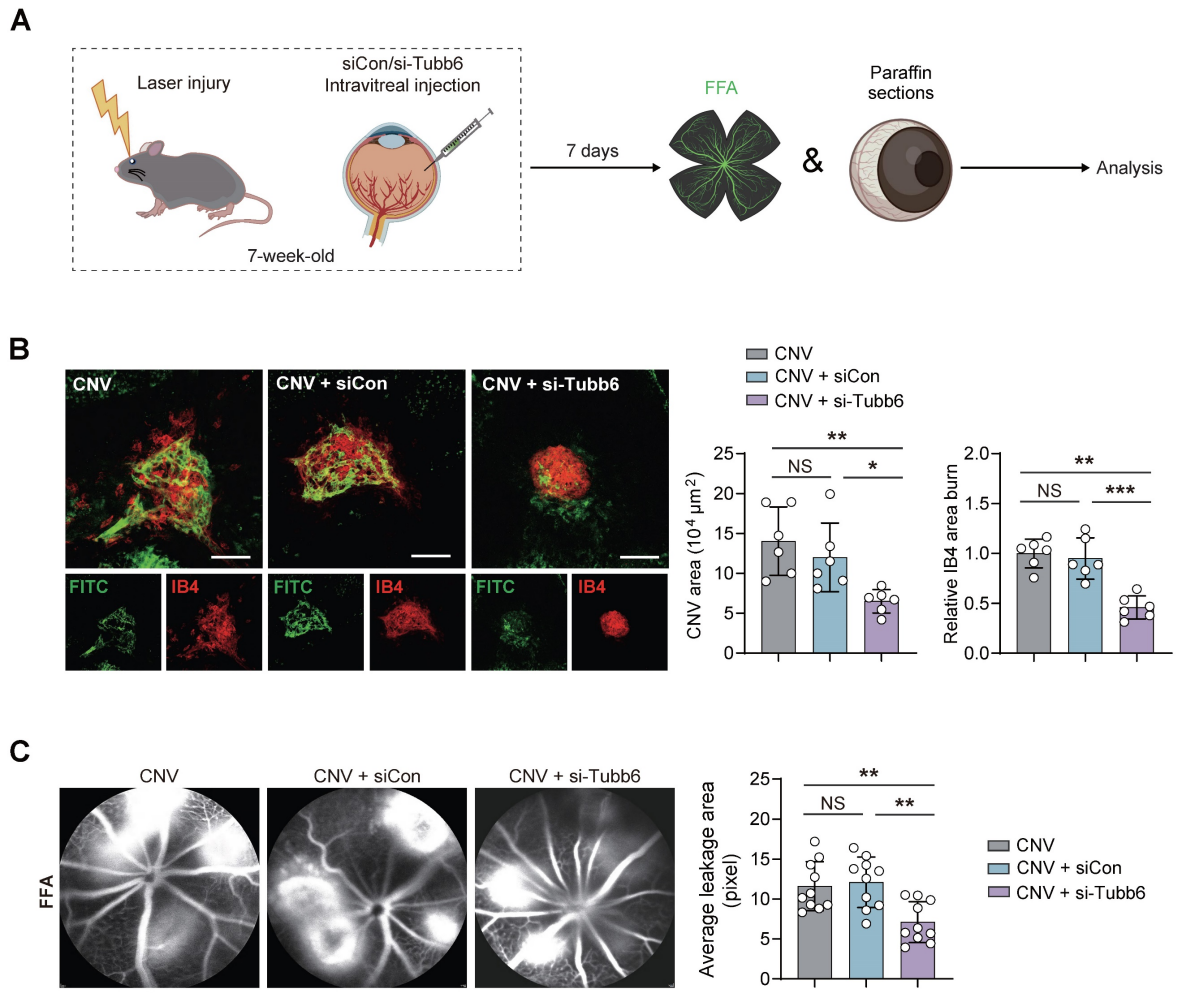
** Tubb6 deficiency inhibits vascular leakage and CNV progression* in vivo*. (A)** Experimental scheme for** (B-C)**. **(B)** FITC-dextran perfusion and IB4 fluorescence of RPE-choroid complexes from laser-induced CNV mice and CNV mice injected with siCon/si-Tubb6. *n* = 6 per group. Scale bar: 100 μm. **(C)** FFA images of laser-induced CNV mice and CNV mice injected with siCon/si-Tubb6. *n* = 10 per group. Data represent different numbers (*n*) of biological replicates. Data are shown as mean ± SD. One-way ANOVA followed by Bonferroni's test is used in **B-C**. NS: not significant (p > 0.05); *p < 0.05; **p < 0.01; and ***p < 0.001.

The impact of Tubb6 on CNV was further explored using mice with laser-induced CNV. Intravitreal injections of siCon and si-Tubb6 were delivered to 7-week-old mice subjected to laser injury, and RPE-choroid complexes were harvested 7 days post-injection** (Figure 7A)**. We used FITC-dextran perfusion and IB4 immunofluorescence staining to assess the impact of Tubb6 on choroidal neovascularization. Compared to the control group, Tubb6-knockdown mice exhibited reduced FITC-dextran leakage, along with a decrease in the IB4-labeled CNV area **(Figure 7B)**. Fluorescence fundus angiography (FFA) demonstrated diminished leakage area in mice intravitreal-injected with si-Tubb6 **(Figure 7C)**, suggesting that Tubb6 deficiency contributed to the restoration of pathological choroid neovascularization and reduced vascular leakage *in vivo*.

### YBX1 drives TUBB6 expression and regulates endothelial functions by enhancing mRNA stability

**Figure 8:**
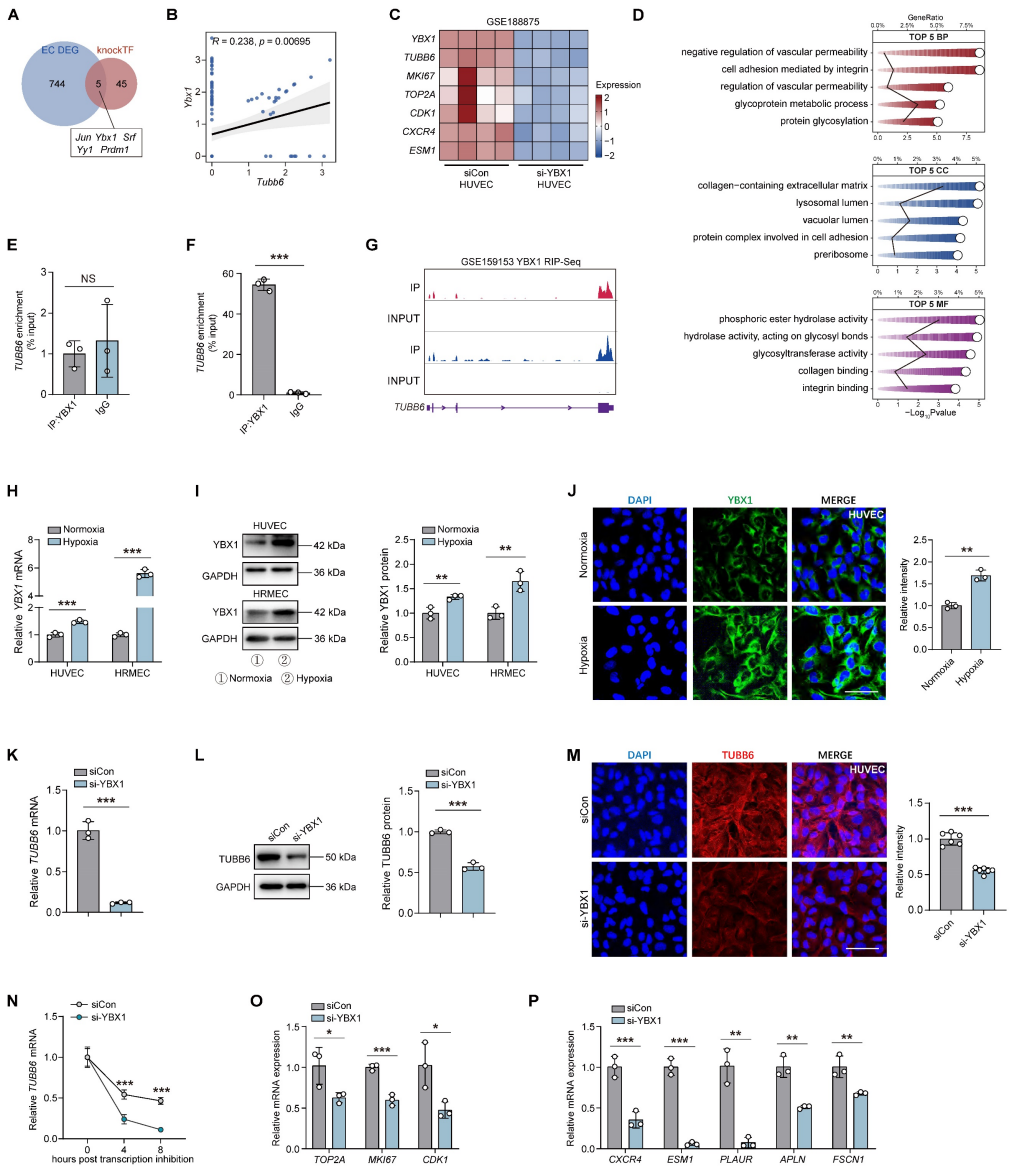
** YBX1 drives TUBB6 expression and regulates endothelial functions by enhancing mRNA stability. (A)** Venn diagram of upregulated DEGs from **Figure 1C** and predicted regulators from public database. **(B)** Scatter plot showing the positive correlation between *Ybx1* and *Tubb6*. **(C)** Heatmap showing interested genes in YBX1-knockdown HUVECs (GEO accession: GSE188875). *n* = 3 per group. **(D)** GO analysis of YBX1-knockdown HUVECs. The top five GO terms were visualized. **(E)** CHIP-qPCR analysis between YBX1 and TUBB6 in HUVECs. *n* = 3 per group. **(F)** RIP-qPCR validates the binding between YBX1 protein and *TUBB6* transcripts in HUVECs. *n* = 3 per group. **(G)** RIP-Seq data validates the binding between YBX1 protein and TUBB6 transcript. **(H)** Relative mRNA expression of *YBX1* in the hypoxia-treated HUVECs and HRMECs compared with respective controls. *n* = 3 per group. **(I)** Relative YBX1 protein levels in the hypoxia-treated HUVECs and HRMECs compared with respective controls. *n* = 3 per group. **(J)** Immunofluorescence staining of YBX1 in the hypoxia-treated HUVECs. *n* = 3 per group. Scale bar: 50 μm. **(K)** mRNA expression of *TUBB6* in the YBX1-knockdown HUVECs. *n* = 3 per group. **(L)** Relative TUBB6 protein in the YBX1-knockdown HUVECs. *n* = 3 per group. **(M)** Immunofluorescence staining of TUBB6 in YBX1-knockdown HUVECs. n = 6 per group. Scale bar: 50 μm. **(N)** The *TUBB6* mRNA level was detected by qPCR in the YBX1-knockdown HUVECs at 0, 4 and 8 hours post actinomycin D treatment. *n* = 3 per group. **(O)** mRNA expression of proliferative markers in the YBX1-knockdown HUVECs. *n* = 3 per group. **(P)** mRNA expression of tip cell markers in the YBX1-knockdown HUVECs. *n* = 3 per group. Data represent different numbers (*n*) of biological replicates. Data are shown as mean ± SD. Two-tailed Student's T test is used in **H-P**. NS: not significant (p > 0.05); *p < 0.05; **p < 0.01; and ***p < 0.001.

We subsequently investigated the mechanisms by which endothelial TUBB6 exacerbates vascular lesions. Utilizing the knockTF database (https://bio.liclab.net/KnockTFv2/) for predicting potential upstream binding proteins of TUBB6, we identified Y-box binding protein 1 (YBX1) as a possible regulator. We have analyzed the DEGs of ECs in **Figure 1C**, and *Ybx1* was found to be upregulated in ECs of OIR retina **(Figure 8A)** and positively correlated with *Tubb6* **(Figure 8B)**. To explore the role of YBX1 in pathological angiogenesis, we re-analyzed public datasets GSE188875 (transcriptome of YBX1-siRNA versus scramble siRNA HUVECs, 42), and discovered that *TUBB6* and proliferative or tip cell-associated markers were downregulated upon YBX1 knockdown **(Figure 8C)**. Enrichment of angiogenesis- and adhesion-related pathways was found in gene ontology (GO) analysis **(Figure 8D)**.

Considering YBX1 functions as both a DNA and RNA-binding protein and has been implicated in diverse cellular processes 43-45, we performed both chromatin immunoprecipitation (CHIP)-qPCR assay **(Figure 8E)** and RNA immunoprecipitation (RIP)-qPCR assay **(Figure 8F)** to elucidate the interaction mechanism between YBX1 and TUBB6. Results implicated that YBX1 directly regulates the transcripts of *TUBB6* on a post-transcriptional level **(Figure 8F)**. RIP-Seq further confirmed that YBX1 modulated *TUBB6* expression by binding to its 3' untranslated region (3' UTR). The binding sites between YBX1 and *TUBB6* were visualized in **Figure 8G**. qPCRs, western blots, and immunofluorescent staining revealed that YBX1 expression was elevated in HUVECs and HRMECs upon hypoxia treatment** (Figure 8H-J)**. To validate the *in vitro* function of YBX1, we transfected HUVECs with YBX1 small interfering RNA (si-YBX1) or scramble siRNA (siCon). The silencing efficiency of YBX1-siRNA in ECs was confirmed via qPCR and western blot **(Supplementary Figure S1C-D)**. qPCRs, western blots, and immunofluorescent staining revealed a significant reduction in both mRNA and protein levels of TUBB6 following YBX1 knockdown **(Figure 8K-M)**. Since YBX1 plays a vital role in RNA stabilization and transcriptional regulation 46, we further investigated its interaction with *TUBB6* mRNA. A shortened half-life of the *TUBB6* transcripts was observed in YBX1-knockdown HUVECs treated with the transcription inhibitor actinomycin D **(Figure 8N)**, indicating that the loss of YBX1 impairs *TUBB6* mRNA stability in HUVECs. Additionally, YBX1 knockdown led to a decrease in the expression of proliferative **(Figure 8O)** and tip cell markers **(Figure 8P)**, emphasizing its essential role in sprouting angiogenesis. These findings collectively demonstrate that YBX1 promotes TUBB6 expression by regulating mRNA stability on the post-transcriptional level.

### YBX1/TUBB6 axis regulates vascular functions through WNT3A/FZD8 signaling

**Figure 9:**
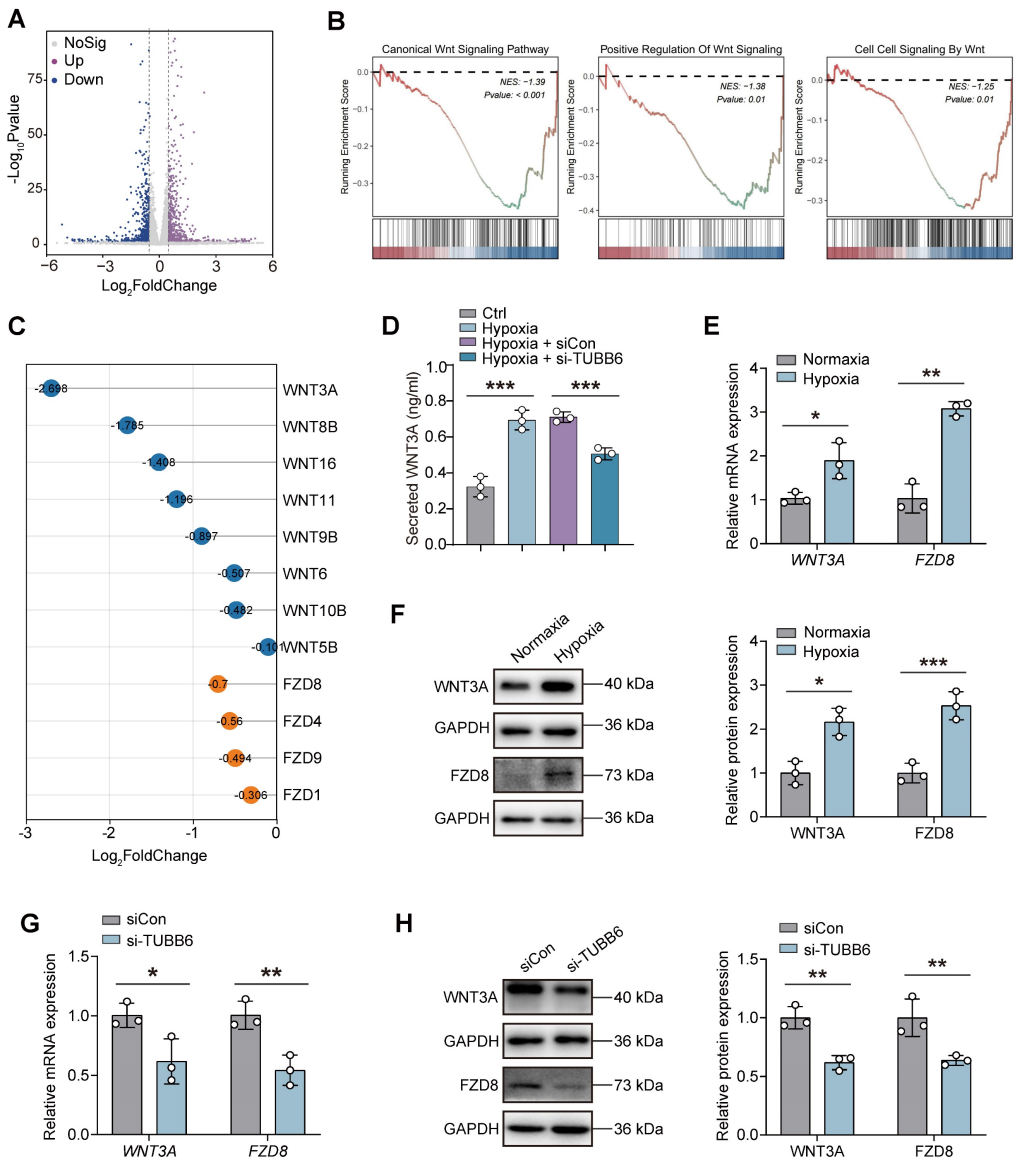
** YBX1/TUBB6 axis regulates vascular functions through WNT3A/FZD8 signaling. (A)** Volcano plots demonstrating DEGs of si-TUBB6 HUVECs vs siCon HUVECs. *n* = 3 per group. **(B)** GSEA plot showing enriched WNT signaling pathways. **(C)** Lollipop chart showing Log_2_Fold Changes of WNT-related genes in the TUBB6-knockdown HUVECs. **(D)** Elisa assay showing secreted WNT3A level in HUVECs with distinct treatment. *n* = 3 per group. **(E)** RNA expression of *WNT3A* and *FZD8* in the hypoxia-treated HUVECs. *n* = 3 per group. **(F)** Protein levels of WNT3A and FZD8 in the hypoxia-treated HUVECs. *n* = 3 per group. **(G)** RNA expression of *WNT3A* and *FZD8* in the TUBB6-knockdown HUVECs. *n* = 3 per group. **(H)** Protein levels of WNT3A and FZD8 in the TUBB6-knockdown HUVECs. *n* = 3 per group. Data represent different numbers (*n*) of biological replicates. Data are shown as mean ± SD. One-way ANOVA followed by Bonferroni's test is used in **D**. Two-tailed Student's T test is used in **E-H**. NS: not significant (p > 0.05); *p < 0.05; **p < 0.01; and ***p < 0.001.

We utilized bulk RNA-Seq to identify downstream targets of TUBB6 in ECs. A total of 645 upregulated genes and 586 downregulated genes (Log_2_ FoldChange > 0.5 or < -0.5; p < 0.05) were identified in HUVECs with TUBB6 knockdown **(Figure 9A)**. Gene set enrichment analysis (GSEA) demonstrated that genes in TUBB6-knockdown HUVECs were most significantly enriched in WNT signaling-related pathways. Multiple WNT-related pathways were suppressed after TUBB6 knockdown **(Figure 9B)**. GSEA analysis also demonstrated multiple activated WNT signaling-related pathways in the ECs from PDR patients in **Figure 1R (Supplementary Figure S5)**. Previous research demonstrated that the inhibition of microtubule dynamics by microtubule-targeting agents (MTAs), such as vinblastine, taxol, and C12 (combretastatin-2-aminoimidazole analog) suppressed Wnt signaling 47. Similarly, PAWI-2, a chemical probe, was observed to inhibit Wnt3a/β-catenin signaling via disturbing microtubule homeostasis 48. Given that TUBB6 is a distinct tubulin isoform and its knockout has been shown to diminish microtubule growth and disrupt the microtubule network 49, which was also confirmed by GSEA analysis in our research **(Supplemental Figure S6)**, we proceeded to examine its downstream targets in HUVECs.

Among the ligands and receptors involved in the WNT signaling pathway, *WNT3A* (Log_2_ Fold Change = -2.698) and *FZD8* (Log_2_ Fold Change = -0.7) showed the most notable Log_2_ Fold Changes following TUBB6 knockdown** (Figure 9C)**. ELISA assays revealed that compared with the control group, an increased level of WNT3A was detected in hypoxia-treated HUVECs, which was rescued by TUBB6 knockdown **(Figure 8D)**. qPCR and western blot analyses confirmed marked increases in WNT3A and FZD8 expression in hypoxic HUVECs versus normal controls** (Figure 9E-F)**. Moreover, RNA and protein expression levels of WNT3A and FZD8 were reduced by TUBB6 knockdown** (Figure 9G-H)**, suggesting that TUBB6 functioned as a pivotal regulator of WNT signaling pathway to modulate vasculature development.

## Discussion

Ocular neovascularization is a prominent pathological feature of several common eye diseases, including retinopathy of prematurity (ROP), proliferative diabetic retinopathy (PDR), and age-related macular degeneration (AMD) 50. While anti-VEGF therapy has become the standard treatment for pathological ocular angiogenesis, a substantial number of patients exhibit insufficient responses, and the efficacy tends to decline over time 51, 52. This indicates that other critical factors might also contribute to ocular angiogenesis beyond the VEGF signaling pathway. We detected up-regulated TUBB6 expression in ECs of PDR patients and nAMD patients, which was also observed in the cultured hypoxic ECs, the retina of OIR mice during the neovascularization stage, as well as the choroid of CNV mice. In the OIR retinas, TUBB6 deficiency rescued disrupted blood-retinal barrier (BRB) and mitigated pathological angiogenesis. In the CNV choroid, TUBB6 reduction reduced vascular leakage and laser-induced CNV area. To better annotate the mechanism of TUBB6 in endothelial activation and sprouting angiogenesis, we further investigate its possible upstream and downstream targets. Mechanistically, YBX1, an RNA-binding protein, was found to directly regulate mRNA stability of *TUBB6* via binding to its 3'UTR in ECs. We also revealed that YBX1-driven TUBB6 upregulation significantly enhanced endothelial cell activation and tip cell formation through modulating WNT3A/FZD8 axis. These findings provide novel insights into the pathogenesis of ocular microvascular diseases and establish a foundation for future therapeutic strategies.

Microtubules are critical components of the eukaryotic cytoskeleton, indispensable for cell division, morphology, motility, and angiogenesis 21, 22. Tubulin dynamic rearrangement is indispensable for vessel sprouting 53, 54. Kobayashi *et al*. reported that detyrosination of α-tubulin in ECs significantly suppressed angiogenesis 22. Retinal stem cells cocultured with OIR-ROP retinal tissues could highly express β-tubulin family and protein kinase C, further promoting angiogenesis 26. Moreover, a range of tubulin inhibitors have demonstrated anti-angiogenic potential 55. Tubulin-associated inhibitors, such as paclitaxel, vinblastine, vincristine, and docetaxel, have long been applied to suppress tumor-associated angiogenesis via interfering with tubulin dynamics 56, 57. TUBB6 plays a key role in the control of microtubule and cytoskeleton dynamics 49. Accumulating evidence suggests that TUBB6, a critical component of microtubule, impacts microtubule dynamics and is closely associated with cell migration and proliferation. Inhibition of TUBB6 could significantly reduce cell migration and invasion ability by suppressing cell cycle progression in bladder cancer 58. Sun *et al*. found that TUBB6 upregulation was beneficial for nerve regeneration after spinal cord injury 59. Additionally, TUBB6 was found to be a migration control factor in non-small cell lung cancer, which posed a negative correlation with patient survival 60. We herein identified the crucial role of TUBB6 in facilitating ocular angiogenesis under pathological conditions, which was required for elucidating the mechanisms of ocular vasculopathy.

We also demonstrated that YBX1 overexpression in hypoxia-treated HUVECs enhanced expression of TUBB6, proliferative-related and tip cell-related genes. A recent study by Yan *et al*. also demonstrated that YBX1 interacted with tsRNA-1599, thus regulating angiogenesis 61. YBX1 has been proven to modulate not only transcription of various genes associated with cell growth, drug resistance and DNA synthesis, but also translation, mRNA stabilization and DNA repair/self-defense processes 62. YBX1 dysregulation contributes to various oculopathies and participates in multiple pathogenesis. Simpson *et al*. found that YBX1 could induce pathological angiogenesis via secretion of angiogenic factors (TGF-β, CSF-1, NGF, ADAM9 and ADAM17) 63. A recent study showed that YBX1 could interact with ZO-1 to regulate endothelial responses during angiogenesis 64. YBX1 was identified as one of top hub genes which participated in type 1 diabetes mellitus (T1DM) 65. YBX1 deficiency could inhibit glucosuria and subnephrotic albuminuria by translationally repressing *Sglt2* transcripts 66. YBX1 also interacted with multiple signaling pathways, including JAK pathway, Wnt pathway, and AKT signaling pathway 67-69. These findings suggest the critical and extensive involvements of YBX1 regulation in retinal vasculopathy. The present study revealed the pivotal role of YBX1 in ocular vascular sprouting, highlighting its necessity for vascular development and the prevention of aberrant vascular phenotypes.

We demonstrated TUBB6 could mediate microvascular anomalies via Wnt signaling pathway. The Wnt signaling pathway is crucial in vascular morphogenesis across various organs, including the eye. Wnt ligands and receptors are key regulators of ocular angiogenesis during eye development and in ocular vasculopathy 70, 71. Wnt signaling was believed to maintain retinal vascularization, blood-brain barrier and blood-retinal barrier 72. The activation of Wnt pathway contributes to the pathogenesis of diabetic retinopathy by exacerbating oxidative stress. Dickkopf homolog 1, a specific Wnt pathway inhibitor, reduced retinal inflammation, vascular leakage, and retinal neovascularization in streptozotocin (STZ)-induced diabetic mice 73. Wnt inhibitor factor 1 (WIF1), a secreted Wnt antagonist, significantly reduced neovascularization and photoreceptor injury in the OIR model by inhibiting the Wnt/β-catenin-VEGF signaling pathway 74. Moreover, previous research showed that MTAs like vinblastine, taxol, and C12 suppressed Wnt signaling by inhibiting microtubule dynamics 47. Similarly, PAWI-2 disrupted microtubule homeostasis, further inhibiting Wnt3a/β-catenin signaling 48. A recent study by Liu *et al.* showed that the novel thiophene derivative, compound 1312, has demonstrated therapeutic efficacy against gastrointestinal cancer by acting as a dual inhibitor of β-tubulin and the Wnt signaling pathway 75. However, the interactions between tubulin proteins and Wnt-related networks are complex and other potential regulatory mechanisms of TUBB6 in ECs might also exist. More comprehensive and in-depth investigations on the endothelial-expressed TUBB6 in ischemic retinopathy are warranted.

## Conclusions

Herein, the combination analysis of scRNA-Seq and bulk RNA-Seq demonstrated a significant elevation of TUBB6 in ECs during pathological ocular angiogenesis. Our research highlights the significance of TUBB6 as a potential therapeutic target and its distribution pattern in proliferative ECs and tip cells. Targeting TUBB6 may aid in developing new strategies to enhance the prognosis for patients who poorly respond to anti-VEGF therapy. Subsequent investigations are essential to explore the interplay between TUBB6 and other signaling pathways to thoroughly clarify its role in ocular pathological angiogenesis.

## Materials and Methods

***Animal experiment.*** All experiments were conducted according to the guidelines of the Association for Research in Vision and Ophthalmology (ARVO) Statement for the Use of Animals in Ophthalmic and Vision Research and approved by the Institutional Animal Care and Use Committee of the authors' institute. C57BL/6J mice (strain ID: 219) were purchased from Charles River Laboratories (Wilmington, MA, USA). All animal experiments were approved and consistently reviewed by the Ethical Review Committee of Nanjing Medical University (approval number: IACUC-2408075). Mice were bred in specific pathogen-free facilities at Nanjing Medical University with a 12 hours light-dark cycle at 28.5 °C, fed with standard laboratory chow and allowed free access to water.

***Oxygen-induced retinopathy (OIR) model.*** The OIR model was established with C57BL/6J mice on postnatal day 7 (P7). On P7, the pups along with their nursing mothers were exposed to 75% oxygen for a duration of 5 days. At P12, they were removed from the oxygen-rich environment and returned to normoxia until P17. Concurrently, age-matched control animals were maintained in room air. Intravitreal injections were administered using a 5 µL Hamilton syringe (Hamilton) fitted with a 33-gauge needle.

***Laser-induced choroid neovascularization (CNV) model.*** Laser photocoagulation of the murine fundus was executed utilizing an argon laser (Lumenis, Inc., Santa Clara, CA, USA) with a central wavelength of 532 nm, an energy delivery of 200 mW, a spot diameter of 100 μm, and a pulse duration of 100 ms to induce Bruch's membrane rupture, thereby establishing a model of laser-induced choroidal neovascularization (CNV). Intravitreal injections were administered via a 5 µL Hamilton syringe (Hamilton, Bonaduz, Switzerland) equipped with a 33-gauge needle.

***Mounting of mice retina and RPE-choroid-sclera complex.*** Mice underwent anesthesia and euthanasia, after which the eyeballs were excised and the connective tissues were trimmed away. For the preparation of retinal flat mounts, the anterior segment of the eye and the vitreous body were meticulously clipped, and the eyeballs were then immersed in 4% paraformaldehyde (PFA) at room temperature for 1 hour for fixation. Following PBS washes, the neural retina was carefully dissected from the posterior eye cup and shaped into four-leaf clover retinal flat mounts for further analyses. For RPE-choroid-sclera complex flat mounts, the entire eye was fixed in 4% PFA at 4°C overnight. After washing with Phosphate-buffered saline (PBS) buffer solution, the anterior segment of the eye and the vitreous body were precisely excised. The RPE-choroid-sclera complex was then isolated and shaped into four-leaf clover flat mounts. For qPCR and immunoblotting analyses, the neural retina or the RPE-choroid-sclera complex was isolated and prepared for RNA and protein extraction.

***Hypoxyprobe staining.*** The hypoxic condition of the retina was evaluated using the Hypoxyprobe RedAPC Kit (Hypoxyprobe, Inc.) following the manufacturer's guidelines. Oxygen-sensitive probes were administered intraperitoneally into postnatal day 17 (P17) OIR mice at a dose of 2.5 mg each, one hour before sampling. The tissues were incubated in a blocking solution composed of 3% bovine serum albumin (BSA) and 0.3% Triton X-100 for one hour, followed by overnight incubation with RED APC dye-MAb1 (1:100, Hypoxyprobe) and Isolectin B4 (1:100, Vector Laboratories). After washing with PBS, the sections were mounted on slides. Fluorescence was examined using an inverted microscope (DMi8, Leica) equipped with a THUNDER imaging system (Leica). The fluorescence results were analyzed using ImageJ software.

***FITC-Dextran evaluating choroidal neovascularization.*** Seven days after the induction of CNV, 0.5 mL of FITC-dextran (average molecular weight: 2000 kDa) was perfused into the hearts of anesthetized mice, which were subsequently euthanized. After the eyeballs were removed and fixed in 4% PFA at 4°C overnight, the RPE-choroid-sclera complex was isolated, and flat mounts were prepared. The tissues were incubated in a blocking solution consisting of 3% BSA and 0.3% Triton X-100 for 1 hour, followed by overnight incubation with Isolectin B4 (1:100, Vector Laboratories). After washing with PBS, the sections were mounted on slides and photographed using a confocal microscope (Carl Zeiss Jena, Germany). The fluorescence results were evaluated using ImageJ software.

***Cryosection of mouse eyes.*** Mouse eyeballs were excised and fixed in FAS fixative for eyes (Servicebio) at 4°C for 1 hour. After PBS washing, they were subjected to gradient dehydration in 10%, 20%, and 30% sucrose solutions, with an overnight stay in 30% sucrose at 4°C. The eyeballs were then embedded in an optimal cutting temperature compound (Sakura) and frozen at -80°C. Sagittal cryosections of 10 µm thickness were subsequently prepared and affixed to slides.

***Ophthalmic evaluations.*** 7 days post-CNV induction, mice were anesthetized, and pupil dilation was performed for fundus examination. Fluorescein fundus angiography (FFA) was conducted after intraperitoneal injection of sodium fluorescein (International Medication Systems) at 2 µL per gram of body weight. Fundus images were captured using the Heidelberg Retinal Angiography 2 system (Heidelberg Engineering).

***Cell culture.*** In this research, we utilized two distinct types of endothelial cells: human umbilical vein endothelial cells (HUVECs; accession number: PCS-100-013; ATCC) and human retinal microvascular endothelial cells (HRMECs; accession number: ACBRI-181). ECs were cultured in DMEM/Low Glucose medium supplemented with 10% fetal bovine serum (FBS; Invitrogen, Carlsbad, CA, USA), penicillin (100U/mL; Invitrogen) and streptomycin (100 U/mL; Invitrogen). Cells were maintained at 37°C with 21% O_2_ and 5% CO_2_. The working concentration of recombinant human VEGF165 (Peprotech, Thermo Fisher Scientific) used for treating HUVECs in **Figure 4** was 20 ng/ml. Additionally, we used 1% FBS to culture HUVECs in scratch, transwell, and tube formation assays to eliminate the interference from cell proliferation. In the actinomycin D assay, HUVECs were cultured in complete medium containing actinomycin D (5 µg/mL) and harvested at 0, 4, and 8 hours post-treatment.

***Cell transfection.*** ECs were transfected with indicated siRNAs using Lipofectamine 3000 (Invitrogen). Scramble siRNA and siRNAs targeting TUBB6 were purchased from RiboBio (Guangzhou, China) and those targeting YBX1 were purchased from GenePharma (Shanghai, China). Specific sequences of these siRNAs were listed in **Supplementary Table S1**.

***TUBB6 knockout (KO) HUVECs.*** TUBB6-knockout HUVECs were established using the CRISPR/Cas9 system. A small guide RNA (sgRNA) targeting the second exon of human TUBB6 gene was designed by Genechem (Shanghai, China) and related sgRNA sequence was supplied in **Supplementary Table S1**. HUVECs were transduced with the lentivirus-packed CRISPR/Cas9-TUBB6-KO construct to form TUBB6-knockout HUVECs and the control group was transduced with lentivirus-packed CRISPR/Cas9-KO construct. HUVEC were then selected with puromycin and dispensed into 96-well plates. The knockout effect of CRISPR/Cas9 system was validated in **Figure S3**.

***RNA isolation and qPCR.*** Total RNA was extracted from cell lysates using the TRIzol reagent (Invitrogen). RNA concentration and purity were quantified using a Nano-Drop ND-1000 spectrophotometer (Nano-Drop Technologies, Wilmington, DE, USA). Complementary DNA (cDNA) was synthesized employing a PrimeScript RT Kit (Takara, Otsu, Shiga, Japan). RNA expression levels were evaluated through quantitative PCR (qPCR) with the FastStart Universal SYBR Green Master (ROX; Roche, Basel, Switzerland) on a StepOne Plus Real-Time PCR System (Applied Biosystems, Darmstadt, Germany). mRNA levels of β-actin were simultaneously measured for normalization purposes. Primer information is detailed in **Supplementary Table S2**.

***Immunoblotting.*** Collected cells were initially lysed in lysis buffer (Beyotime, Shanghai, China) containing a protease inhibitor cocktail (Roche) for protein extraction. The isolated proteins were subsequently fractionated by size using sodium dodecyl sulfate-polyacrylamide gel electrophoresis (SDS-PAGE) and transferred onto a polyvinylidene fluoride (PVDF) membrane (Millipore, Boston, MA, USA) via a wet transfer system. Membranes were then blocked with bovine serum albumin (BSA) and sequentially incubated with the relevant primary antibodies (specified in **Supplementary Table S3**) and HRP-conjugated secondary antibodies (dilution: 1:10000; ICL).

***Immunofluorescence staining.*** Cells, eye cryosections, and retinal flat mounts were incubated in a blocking solution containing 3% BSA and 0.3% Triton X-100 for one hour. Subsequently, the samples were incubated overnight at 4°C with primary antibodies **(Supplementary Table S3)**, followed by incubation with fluorescence-conjugated secondary antibodies (1:500) for two hours at room temperature. DAPI (Sigma-Aldrich, St. Louis, MO, USA) was utilized for nuclear counterstaining. Fluorescence observations were made using an inverted microscope (DMi8, Leica, Wetzlar, Germany) equipped with a THUNDER imaging system (Leica). Quantification of fluorescence intensity was performed using ImageJ software.

***Cell cycle analysis.*** Cell cycle progression was evaluated using the Cell Cycle Staining Kit (Multisciences Biotech, Hangzhou, China) in accordance with the manufacturer's protocol. Harvested cells were incubated in a DNA staining solution containing an osmotic agent for 30 minutes at ambient temperature, shielded from light. The cell cycle was interrogated via flow cytometry (Beckman Coulter, Brea, CA, USA), and the resulting data were processed using FlowJo software. The analysis included a total of 10,000 cells per sample.

***EdU assay.*** Cell proliferation was determined using the EdU Apollo567 Kit (RiboBio) following the manufacturer's guidelines. Cells underwent a 2-hour incubation in EdU solution, then fixation with 4% paraformaldehyde (PFA), permeabilization with 0.5% Triton X-100, and subsequent fluorescent labeling in Apollo solution. Proliferating cells with red fluorescence were visualized using an inverted microscope (DMi8) with a THUNDER imaging system.

***Transwell migration assay.*** The transwell chambers with an 8 μm pore size were used to assess cell migration. Treated cells were placed in the upper chamber containing medium and allowed to migrate for 24 hours under conditions of 37°C, 21% O_2_, and 5% CO_2_. Cells that migrated to the lower chamber were collected and then fixed with crystal violet (Beyotime). Five random fields per chamber were examined and averaged using the ECLIPSE Ts2 inverted microscope (Nikon, Shanghai, China).

***Scratch assay.*** A straight line was scratched on the HUVECs with a pipette tip to create a wound. After gently washing with PBS to remove cell debris, the medium was added. A line was drawn at the edge of the well to mark the position. Images of the same scratch area were captured immediately after scratching and 24 hours later using the ECLIPSE Ts2 inverted microscope. Data were analyzed using ImageJ software.

***Tube formation assay.*** HUVECs were planted on growth factor-reduced Matrigel (BD Biosciences) in 24-well plates at a density of 1 × 10^5^ cells per well to monitor the development of capillary-like structures. Images were captured 5 hours after seeding using an ECLIPSE Ts2 inverted microscope. ImageJ software was utilized to measure the number of nodes and the length of branching of the HUVECs.

***ELISA.*** The culture medium of HUVECs was gathered to measure WNT3A secretion using a commercial human WNT3A ELISA kit (CSB-EL026136HU-48T; Biocompare) according to the manufacturer's instructions. The absorbance at 450 nm was measured using a Multiskan FC spectrophotometric plate reader (Thermo Fisher Scientific).

***CHIP-qPCR.*** Chromatin Immunoprecipitation followed by quantitative PCR (ChIP-qPCR) was conducted following the protocols outlined in the SimpleChIP® Enzymatic Chromatin IP Kit (Cell Signaling Technology). Cells were treated with 1% formaldehyde for cross-linking, subsequently sonicated, and enzymatically digested to yield chromatin fragments of suitable size. These DNA fragments were then isolated and incubated overnight at 4°C with an antibody against YBX1. Post incubation, qPCR was utilized to evaluate the DNA fragments enriched for YBX1 binding, with results normalized against the input controls for analysis.

***RIP-qPCR.*** The RIP experiment utilized the Magna RIP Kit (Millipore) following the manufacturer's instructions. In summary, HUVECs were treated with RIP lysis buffer to dissociate RNA-protein complexes. Ten percent of the RIP lysate was reserved as an input reference. For immunoprecipitation, protein A/G magnetic beads were sequentially incubated with either IgG or YBX1 antibody and the cell lysates. The magnetic separator then isolated the magnetic bead-bound complexes using a magnet, capturing the specific RNA-protein complex. The RNA isolated from these complexes was subsequently purified for cDNA synthesis and analyzed via qPCR, with results normalized against the input control.

***Bulk RNA-Seq and analysis.*** Total RNA was isolated from cell lysates using TRIzol reagent ((Invitrogen, Carlsbad, CA, USA). The concentration and purity of RNA were measured with a Nano-Drop ND-1000 spectrophotometer (Nano-Drop Technologies, Wilmington, DE, USA) and the Agilent 2100 Bioanalyzer system (Agilent BioTek; Winooski, VT, USA). RNA with high quality was sent for library construction following the manufacturer's recommendations. The RNA-Seq library was further sequenced with Illumina Novaseq 6000 platform (Illumina) to generate 150 base pair (bp) reads. Then, HTSeq software (version 0.9.1) was used to get the raw count. Quality control and raw sequencing data filtration were achieved with the Fastp (version 0.23.2) and sortmerna (version 4.3.4) software. The STAR software (version 2.7.10) was then applied to align clean reads to the human genome GRCh38. The Salmon v1.9.0 was used to calculate counts and transcript per million reads (TPM) of each gene. DESeq2 (version 1.34.0) was applied to identify differentially expressed genes. Results with p < 0.05 are considered statistically significant. Pathway enrichment analyses were performed based on the differentially expressed genes. The RNA-Seq data has been deposited in GEO database under the accession number GSE275653.

***ScRNA-Seq and analysis.*** Single cell data was analyzed using R package Seurat (version 4.3.0) for dimension-reduction and clustering. OIR and control datasets were integrated with R package Harmony (version 1.2.0) to remove batch effects. Differential expression analysis was performed with FindMarkers or FindAllMarkers functions. R package Monocle2 (version 2.28.0) and slingshot (version 2.12.0) was used to predict a pseudotime trajectory of endothelial activation in the OIR retina. Endothelial cells were ordered in pseudotime with DDRTree and orderCells functions. Vlnplots was visualized with R package dittoSeq (version 1.12.1).

***GO ontology analysis.*** Differential expression genes (DEGs) from bulk RNA-Seq were used for GO analysis with R package clusterProfiler (version 4.8.3). Enriched GO pathways with p < 0.05 were considered statistically significant.

***Correlation analyses.*** For correlation analysis, the Pearson correlation between interested genes was calculated with R package stats (version 4.3.1) and visualized using Chiplot (https://www.chiplot.online/).

***Statistical analysis.*** All data were reported as mean ± SD. Statistical analysis was conducted utilizing GraphPad Prism 10.1.2. Significant differences were assessed via a Two-tailed Student's T test for two-group comparisons, or one-way ANOVA for multiple group comparisons. Results with p < 0.05 are considered statistically significant.

## Supplementary Material

Supplementary figures and tables.

## Abbreviations

Ang-1: angiogenin-1
CNS: central nervous system
CNV: choroidal neovascularization
DEGs: differentially expressed genes
ECs: endothelial cells
FFA: fundus angiography
HUVEC: human umbilical vein endothelial cells
HRMECs: human retinal microvascular endothelial cells
IGF-1: insulin-like growth factor-1
nAMD: neovascular age-related macular degeneration
NVT: neovascular tufts
OIR: oxygen-induced retinopathy
PDR: proliferative diabetic retinopathy
PKC: protein kinase C
RGCs: retinal ganglion cells
ROP: retinopathy of prematurity
scRNA-Seq: single-cell RNA sequencing
sgRNA: small guide RNA
STZ: streptozotocin
TUBB6: tubulin beta-6 chain
T1DM: type 1 diabetes mellitus
VE-cadherin: vascular-endothelial-specific cadherin
VEGF: vascular endothelial growth factors
WIF1: Wnt inhibitor factor 1
YBX1: Y-box binding protein 1
3'UTR: 3' untranslated region

## References

[1] Goveia J, Rohlenova K, Taverna F, Treps L, Conradi LC, Pircher A (2020). An Integrated Gene Expression Landscape Profiling Approach to Identify Lung Tumor Endothelial Cell Heterogeneity and Angiogenic Candidates. Cancer Cell.

[2] Rohlenova K, Goveia J, García-Caballero M, Subramanian A, Kalucka J, Treps L (2020). Single-Cell RNA Sequencing Maps Endothelial Metabolic Plasticity in Pathological Angiogenesis. Cell Metab.

[3] Zarkada G, Howard JP, Xiao X, Park H, Bizou M, Leclerc S (2021). Specialized endothelial tip cells guide neuroretina vascularization and blood-retina-barrier formation. Dev Cell.

[4] Gerhardt H, Golding M, Fruttiger M, Ruhrberg C, Lundkvist A, Abramsson A (2003). VEGF guides angiogenic sprouting utilizing endothelial tip cell filopodia. J Cell Biol.

[5] Turati M, Mattei G, Boaretto A, Magi A, Calvani M, Ronca R (2023). Molecular Profiling of Lymphatic Endothelial Cell Activation In Vitro. Int J Mol Sci.

[6] Herklotz M, Hanke J, Hänsel S, Drichel J, Marx M, Maitz MF (2016). Biomaterials trigger endothelial cell activation when co-incubated with human whole blood. Biomaterials.

[7] Bhakuni T, Norden PR, Ujiie N, Tan C, Lee SK, Tedeschi T (2024). FOXC1 regulates endothelial CD98 (LAT1/4F2hc) expression in retinal angiogenesis and blood-retina barrier formation. Nat Commun.

[8] Pran Babu SPS, White D, Corson TW (2020). Ferrochelatase regulates retinal neovascularization. FASEB J.

[9] Regula JT, Lundh von Leithner P, Foxton R, Barathi VA, Cheung CM, Bo Tun SB (2016). Targeting key angiogenic pathways with a bispecific CrossMAb optimized for neovascular eye diseases. EMBO Mol Med.

[10] Yang S, Li T, Jia H, Gao M, Li Y, Wan X (2022). Targeting C3b/C4b and VEGF with a bispecific fusion protein optimized for neovascular age-related macular degeneration therapy. Sci Transl Med.

[11] Xu M, Fan R, Fan X, Shao Y, Li X (2022). Progress and Challenges of Anti-VEGF Agents and Their Sustained-Release Strategies for Retinal Angiogenesis. Drug Des Devel Ther.

[12] Ricci F, Bandello F, Navarra P, Staurenghi G, Stumpp M, Zarbin M (2020). Neovascular Age-Related Macular Degeneration: Therapeutic Management and New-Upcoming Approaches. Int J Mol Sci.

[13] Zehden JA, Mortensen XM, Reddy A, Zhang AY (2022). Systemic and Ocular Adverse Events with Intravitreal Anti-VEGF Therapy Used in the Treatment of Diabetic Retinopathy: a Review. Curr Diab Rep.

[14] Ribatti D, Solimando AG, Pezzella F (2021). The Anti-VEGF(R) Drug Discovery Legacy: Improving Attrition Rates by Breaking the Vicious Cycle of Angiogenesis in Cancer. Cancers (Basel).

[15] Bai W, Ren JS, Xia M, Zhao Y, Ding JJ, Chen X (2023). Targeting FSCN1 with an oral small-molecule inhibitor for treating ocular neovascularization. J Transl Med.

[16] Yang S, Zhao J, Sun X (2016). Resistance to anti-VEGF therapy in neovascular age-related macular degeneration: a comprehensive review. Drug Des Devel Ther.

[17] Yorston D (2014). Anti-VEGF drugs in the prevention of blindness. Community Eye Health.

[18] Chen D, Shao M, Song Y, Ren G, Guo F, Fan X (2023). Single-cell RNA-seq with spatial transcriptomics to create an atlas of human diabetic kidney disease. FASEB J.

[19] Xia M, Jiao L, Wang XH, Tong M, Yao MD, Li XM (2023). Single-cell RNA sequencing reveals a unique pericyte type associated with capillary dysfunction. Theranostics.

[20] Zhang T, Zhao F, Lin Y, Liu M, Zhou H, Cui F (2024). Integrated analysis of single-cell and bulk transcriptomics develops a robust neuroendocrine cell-intrinsic signature to predict prostate cancer progression. Theranostics.

[21] Liu Z, Huang L, Zhou T, Chang X, Yang Y, Shi Y (2022). A novel tubulin inhibitor, 6h, suppresses tumor-associated angiogenesis and shows potent antitumor activity against non-small cell lung cancers. J Biol Chem.

[22] Kobayashi M, Wakabayashi I, Suzuki Y, Fujiwara K, Nakayama M, Watabe T (2021). Tubulin carboxypeptidase activity of vasohibin-1 inhibits angiogenesis by interfering with endocytosis and trafficking of pro-angiogenic factor receptors. Angiogenesis.

[23] Janke C, Magiera MM (2020). The tubulin code and its role in controlling microtubule properties and functions. Nat Rev Mol Cell Biol.

[24] Bai CW, Lu L, Zhang JN, Zhou C, Ni YC, Li KR (2024). G protein subunit alpha i2's pivotal role in angiogenesis. Theranostics.

[25] Prokosch V, Brockhaus K, Anders F, Liu H, Mercieca K, Gericke A (2020). Elevated intraocular pressure induces neuron-specific β-III-tubulin expression in non-neuronal vascular cells. Acta Ophthalmol.

[26] Ma QQ, Liu FY, Shi M, Sun CH, Tan Z, Chang XD (2019). Bone marrow mesenchymal stem cells modified by angiogenin-1 promotes tissue repair in mice with oxygen-induced retinopathy of prematurity by promoting retinal stem cell proliferation and differentiation. J Cell Physiol.

[27] Binet F, Cagnone G, Crespo-Garcia S, Hata M, Neault M, Dejda A (2020). Neutrophil extracellular traps target senescent vasculature for tissue remodeling in retinopathy. Science.

[28] Pauleikhoff L, Boneva S, Boeck M, Schlecht A, Schlunck G, Agostini H (2023). Transcriptional Comparison of Human and Murine Retinal Neovascularization. Invest Ophthalmol Vis Sci.

[29] Lam JD, Oh DJ, Wong LL, Amarnani D, Park-Windhol C, Sanchez AV (2017). Identification of RUNX1 as a Mediator of Aberrant Retinal Angiogenesis. Diabetes.

[30] Liu YS, Pan JQ, Pan XB, Kong FS, Zhang JQ, Wei ZY (2024). Comparative Analysis of Molecular Landscape in Mouse Models and Patients Reveals Conserved Inflammation Pathways in Age-Related Macular Degeneration. Invest Ophthalmol Vis Sci.

[31] Voigt AP, Mulfaul K, Mullin NK, Flamme-Wiese MJ, Giacalone JC, Stone EM (2019). Single-cell transcriptomics of the human retinal pigment epithelium and choroid in health and macular degeneration. Proc Natl Acad Sci U S A.

[32] Watson EC, Grant ZL, Coultas L (2017). Endothelial cell apoptosis in angiogenesis and vessel regression. Cell Mol Life Sci.

[33] Sabbagh MF, Heng JS, Luo C, Castanon RG, Nery JR, Rattner A (2018). Transcriptional and epigenomic landscapes of CNS and non-CNS vascular endothelial cells. Elife.

[34] Smith TL, Oubaha M, Cagnone G, Boscher C, Kim JS, El Bakkouri Y (2021). eNOS controls angiogenic sprouting and retinal neovascularization through the regulation of endothelial cell polarity. Cell Mol Life Sci.

[35] Connor KM, Krah NM, Dennison RJ, Aderman CM, Chen J, Guerin KI (2009). Quantification of oxygen-induced retinopathy in the mouse: a model of vessel loss, vessel regrowth and pathological angiogenesis. Nat Protoc.

[36] Ma X, Wu W, Liang W, Takahashi Y, Cai J, Ma JX (2022). Modulation of cGAS-STING signaling by PPARα in a mouse model of ischemia-induced retinopathy. Proc Natl Acad Sci U S A.

[37] Selvam S, Kumar T, Fruttiger M (2018). Retinal vasculature development in health and disease. Prog Retin Eye Res.

[38] Alizadeh E, Mammadzada P, André H (2018). The Different Facades of Retinal and Choroidal Endothelial Cells in Response to Hypoxia. Int J Mol Sci.

[39] Yang Z, Ni B, Zhou T, Huang Z, Zhou H, Zhou Y (2023). HIF-1α Reduction by Lowering Intraocular Pressure Alleviated Retinal Neovascularization. Biomolecules.

[40] Vestweber D (2008). VE-cadherin: the major endothelial adhesion molecule controlling cellular junctions and blood vessel formation. Arterioscler Thromb Vasc Biol.

[41] Giannotta M, Trani M, Dejana E (2013). VE-cadherin and endothelial adherens junctions: active guardians of vascular integrity. Dev Cell.

[42] Gross DA, Cheng HS, Zhuang R, McCoy MG, Pérez-Cremades D, Salyers Z (2022). Deficiency of lncRNA SNHG12 impairs ischemic limb neovascularization by altering an endothelial cell cycle pathway. JCI Insight.

[43] Feng M, Xie X, Han G, Zhang T, Li Y, Li Y (2021). YBX1 is required for maintaining myeloid leukemia cell survival by regulating BCL2 stability in an m6A-dependent manner. Blood.

[44] Ni P, Zhou C, Liang S, Jiang Y, Liu D, Shao Z (2023). YBX1-Mediated DNA Methylation-Dependent SHANK3 Expression in PBMCs and Developing Cortical Interneurons in Schizophrenia. Adv Sci (Weinh).

[45] Li J, Zhang B, Feng Z, An D, Zhou Z, Wan C (2024). Stabilization of KPNB1 by deubiquitinase USP7 promotes glioblastoma progression through the YBX1-NLGN3 axis. J Exp Clin Cancer Res.

[46] Wu R, Cao S, Li F, Feng S, Shu G, Wang L (2022). RNA-binding protein YBX1 promotes brown adipogenesis and thermogenesis via PINK1/PRKN-mediated mitophagy. FASEB J.

[47] Kumari A, Shriwas O, Sisodiya S, Santra MK, Guchhait SK, Dash R (2021). Microtubule-targeting agents impair kinesin-2-dependent nuclear transport of β-catenin: Evidence of inhibition of Wnt/β-catenin signaling as an important antitumor mechanism of microtubule-targeting agents. FASEB J.

[48] Cheng J, Tsuda M, Okolotowicz K, Dwyer M, Bushway PJ, Colas AR (2021). Small-molecule probe reveals a kinase cascade that links stress signaling to TCF/LEF and Wnt responsiveness. Cell Chem Biol.

[49] Maurin J, Morel A, Guérit D, Cau J, Urbach S, Blangy A (2021). The Beta-Tubulin Isotype TUBB6 Controls Microtubule and Actin Dynamics in Osteoclasts. Front Cell Dev Biol.

[50] Dorrell M, Uusitalo-Jarvinen H, Aguilar E, Friedlander M (2007). Ocular neovascularization: basic mechanisms and therapeutic advances. Surv Ophthalmol.

[51] Mettu PS, Allingham MJ, Cousins SW (2021). Incomplete response to Anti-VEGF therapy in neovascular AMD: Exploring disease mechanisms and therapeutic opportunities. Prog Retin Eye Res.

[52] Amoaku WM, Chakravarthy U, Gale R, Gavin M, Ghanchi F, Gibson J (2015). Defining response to anti-VEGF therapies in neovascular AMD. Eye (Lond).

[53] Wen L, Yan W, Zhu L, Tang C, Wang G (2023). The role of blood flow in vessel remodeling and its regulatory mechanism during developmental angiogenesis. Cell Mol Life Sci.

[54] Jordan MA, Wilson L (2004). Microtubules as a target for anticancer drugs. Nat Rev Cancer.

[55] Banerjee S, Hwang DJ, Li W, Miller DD (2016). Current Advances of Tubulin Inhibitors in Nanoparticle Drug Delivery and Vascular Disruption/Angiogenesis. Molecules.

[56] Sherbet GV (2017). Suppression of angiogenesis and tumour progression by combretastatin and derivatives. Cancer Lett.

[57] Lu Y, Chen J, Xiao M, Li W, Miller DD (2012). An overview of tubulin inhibitors that interact with the colchicine binding site. Pharm Res.

[58] Kim B, Jung M, Moon KC, Han D, Kim K, Kim H (2023). Quantitative proteomics identifies TUBB6 as a biomarker of muscle-invasion and poor prognosis in bladder cancer. Int J Cancer.

[59] Liu B, Liu G, Li C, Liu S, Sun D (2023). Resection of Scar Tissue in Rats With Spinal Cord Injury Can Promote the Expression of βⅢ-tubulin in the Injured Area. World Neurosurg.

[60] Wen S, Zou R, Du X, Pan R, Li R, Xia J (2024). Identification of macrophage-related genes correlated with prognosis and immunotherapy efficacy in non-small cell lung cancer. Heliyon.

[61] Han XY, Kong LJ, Li D, Tong M, Li XM, Zhao C (2024). Targeting endothelial glycolytic reprogramming by tsRNA-1599 for ocular anti-angiogenesis therapy. Theranostics.

[62] Kuwano M, Uchiumi T, Hayakawa H, Ono M, Wada M, Izumi H (2003). The basic and clinical implications of ABC transporters, Y-box-binding protein-1 (YB-1) and angiogenesis-related factors in human malignancies. Cancer Sci.

[63] Gopal SK, Greening DW, Mathias RA, Ji H, Rai A, Chen M (2015). YBX1/YB-1 induces partial EMT and tumourigenicity through secretion of angiogenic factors into the extracellular microenvironment. Oncotarget.

[64] El Bakkouri Y, Chidiac R, Delisle C, Corriveau J, Cagnone G, Gaonac'h-Lovejoy V (2024). ZO-1 interacts with YB-1 in endothelial cells to regulate stress granule formation during angiogenesis. Nat Commun.

[65] Pujar M, Vastrad B, Kavatagimath S, Vastrad C, Kotturshetti S (2022). Identification of candidate biomarkers and pathways associated with type 1 diabetes mellitus using bioinformatics analysis. Sci Rep.

[66] Bernhardt A, Häberer S, Xu J, Damerau H, Steffen J, Reichardt C (2021). High salt diet-induced proximal tubular phenotypic changes and sodium-glucose cotransporter-2 expression are coordinated by cold shock Y-box binding protein-1. FASEB J.

[67] Shi X, Hu Z, Bai S, Zong C, Xue H, Li Y (2024). YBX1 promotes stemness and cisplatin insensitivity in intrahepatic cholangiocarcinoma via the AKT/β-catenin axis. J Gene Med.

[68] Chao HM, Huang HX, Chang PH, Tseng KC, Miyajima A, Chern E (2017). Y-box binding protein-1 promotes hepatocellular carcinoma-initiating cell progression and tumorigenesis via Wnt/β-catenin pathway. Oncotarget.

[69] Jayavelu AK, Schnöder TM, Perner F, Herzog C, Meiler A, Krishnamoorthy G (2020). Splicing factor YBX1 mediates persistence of JAK2-mutated neoplasms. Nature.

[70] Wang Z, Liu CH, Huang S, Chen J (2019). Wnt Signaling in vascular eye diseases. Prog Retin Eye Res.

[71] Shah R, Amador C, Chun ST, Ghiam S, Saghizadeh M, Kramerov AA (2023). Non-canonical Wnt signaling in the eye. Prog Retin Eye Res.

[72] Drenser KA (2016). Wnt signaling pathway in retinal vascularization. Eye Brain.

[73] Chen Y, Hu Y, Zhou T, Zhou KK, Mott R, Wu M (2009). Activation of the Wnt pathway plays a pathogenic role in diabetic retinopathy in humans and animal models. Am J Pathol.

[74] Tan W, Xu H, Chen B, Duan T, Liu K, Zou J (2022). Wnt inhibitory 1 ameliorates neovascularization and attenuates photoreceptor injury in an oxygen-induced retinopathy mouse model. Biofactors.

[75] Fu L, Li F, Xue X, Xi H, Sun X, Hu R (2024). Exploring the potential of thiophene derivatives as dual inhibitors of β-tubulin and Wnt/β-catenin pathways for gastrointestinal cancers in vitro. Heliyon.

