# Supplementary material for: YBX1-driven TUBB6 upregulation facilitates ocular angiogenesis via WNT3A-FZD8 pathway
Source: Theranostics. 2025 Jan 27;15(7):2680–99. doi: 10.7150/thno.104573 (PMC11898281; doi:10.7150/thno.104573)
Supplement: Supplementary file 1 — Supplementary figures and tables. [file thnov15p2680s1.pdf]

Supplementary Materials

***YBX1*-driven *TUBB6* upregulation facilitates ocular angiogenesis via *WNT3A-FZD8* pathway**

Supplementary materials include 6 Supplementary Figures and 3 Supplementary Tables.

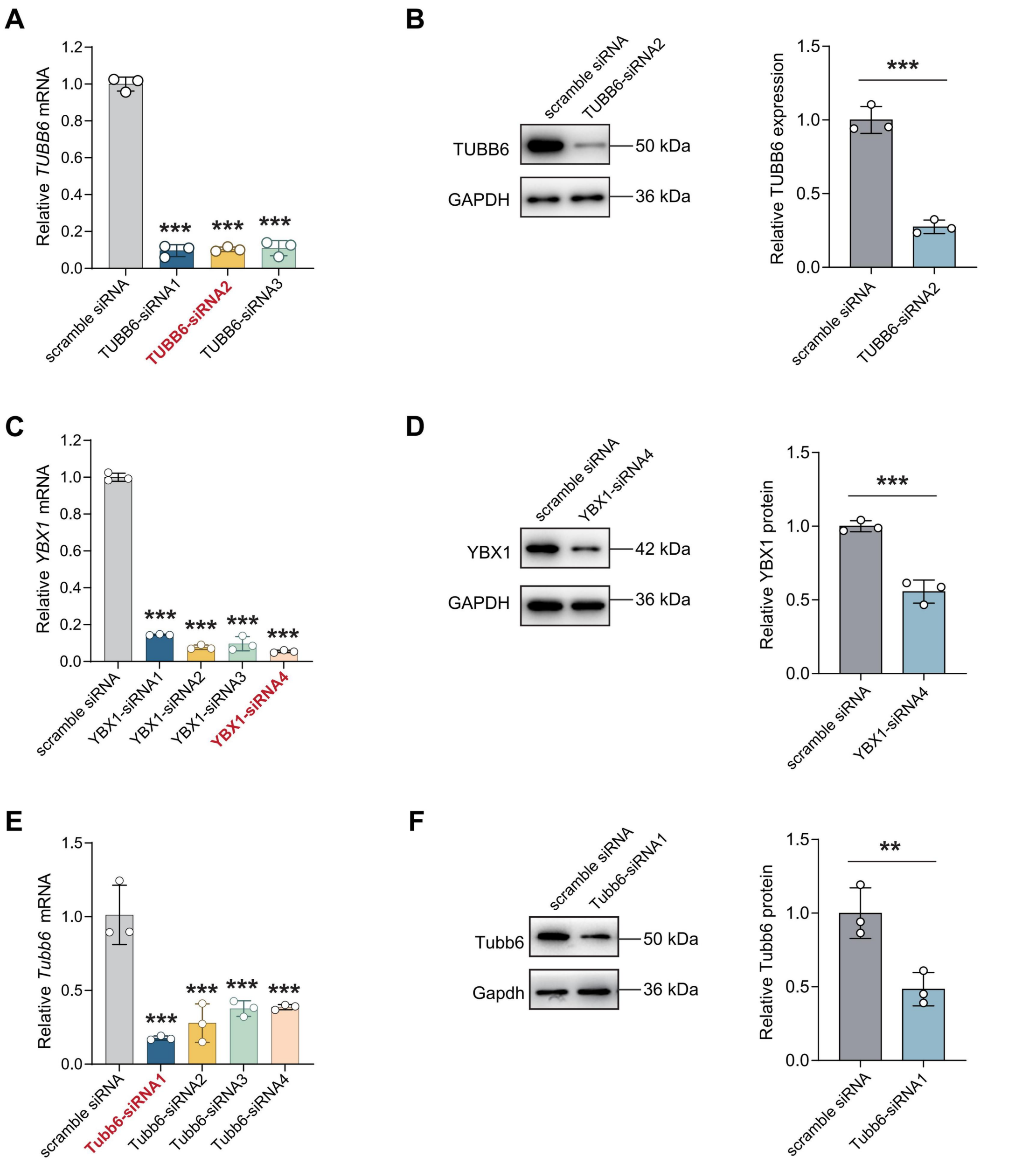

Supplemental Figure S1. Knockdown efficiency of TUBB6 and YBX1 *in vitro* and *in vivo*. (A-B) qPCR **(A)** and immunoblotting **(B)** confirmed TUBB6 downregulation in TUBB6-siRNA-treated HUVECs. *n* = 3 per group. **(C-D)** qPCR **(C)** and immunoblotting **(D)** confirmed YBX1 downregulation in YBX1-siRNA-treated HUVECs. *n* = 3 per group. **(E-F)** qPCR **(E)** and immunoblotting **(F)** confirmed Tubb6 downregulation in retinas of mice injected with Tubb6-siRNA. *n* = 3 per group. Data represent different numbers (*n*) of biological replicates. Data are shown as mean ± SD. Two-tailed Student's T test is used in **A-F**. **p < 0.01 and ***p < 0.001.

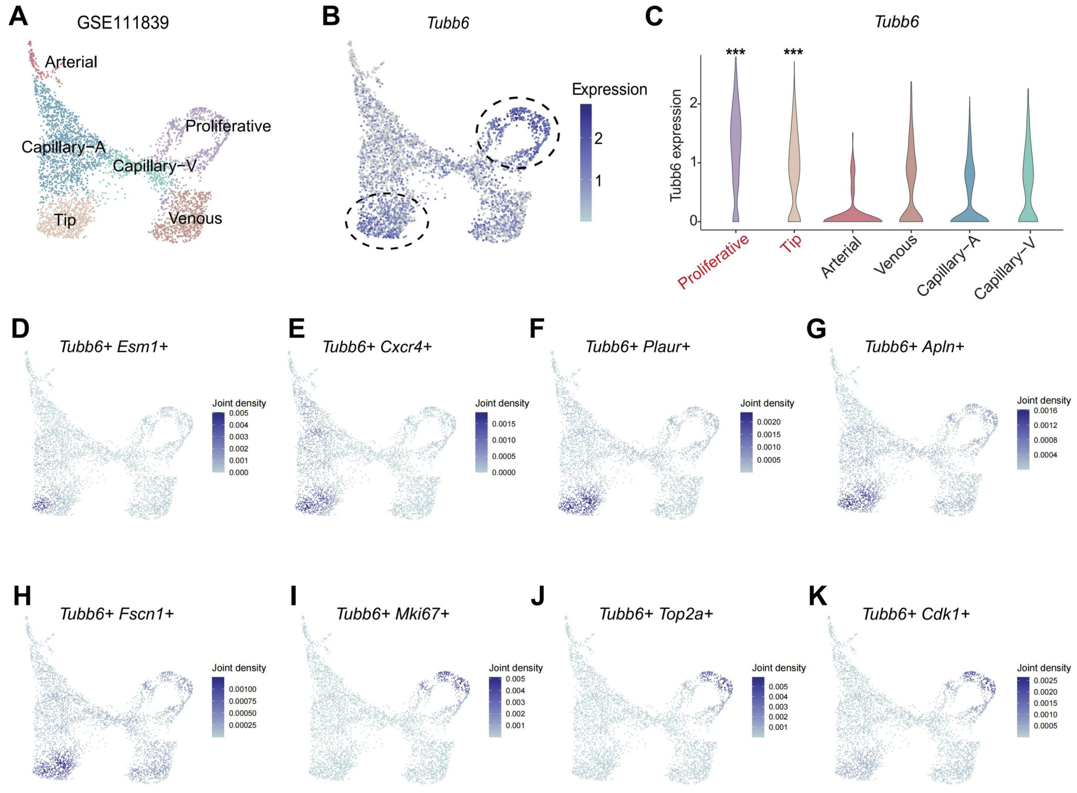

Supplemental Figure S2. Tubb6 expression pattern in postnatal day 7 (P7) ECs from mice brain. (A) UMAP plot of EC subtypes identified in murine brain (GEO accession: GSE111839). **(B-C)** Feature plot **(B)** and violin plot **(C)** showed that *Tubb6* was highly expressed in proliferative ECs and tip cells compared to other EC subtypes. **(D-H)** *Tubb6* was co-expresssed with various tip cell markers in the ECs of murine brain. **(I-K)** *Tubb6* was co-expresssed with multple proliferative markers in the ECs of murine brain. Non-parametric Wilcoxon rank sum test is used in **C**. ***p < 0.001.

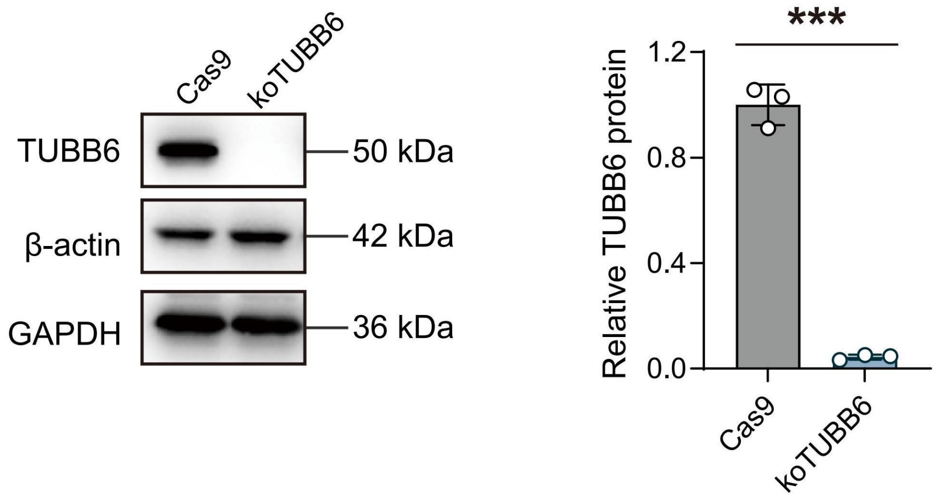

Supplemental Figure S3. TUBB6-knockout HUVECs constructed with the CRISPR/Cas9 system. Immunoblotting confirmed TUBB6 knockout in HUVECs with Cas9-expressing construct plus the CRISPR/Cas9-TUBB6-KO construct. *n* = 3 per group. Data represent different numbers (*n*) of biological replicates. Data are shown as mean ± SD. Two-tailed Student's T test is used. ***p < 0.001.

```
                     1        10        20        30        40        50        60        70
Homo_sapiens         MREIVHIQAGQCGNQIGTKFWEVISDEHGIDQAGGYVGDSALQLERISVYYNESSSKKYVPRAALVDLEP
Mus_musculus         MREIVHIQAGQCGNQIGTKFWEVISDEHGIDQAGGYVGDSALQLERISVYYNESSSKKYVPRAALVDLEP
Rattus_norvegicus    MREIVHIQAGQCGNQIGTKFWEVISDEHGIDPAGGYVGDSALQLERISVYYNESSSKKYVPRAALVDLEP
consensus>70         MREIVHIQAGQCGNQIGTKFWEVISDEHGID.AGGYVGDSALQLERISVYYNESSSKKYVPRAALVDLEP

                              80        90       100       110       120       130       140
Homo_sapiens         GTMDSVRSGPFGQLFRPDNFIFGQTGAGNNWAKGHYTEGAELVDSVLDVVRKECEHCDCLQGFQLTHSLG
Mus_musculus         GTMDSVRSGPFGQLFRPDNFIFGQTGAGNNWAKGHYTEGAELVDSVLDVVRKECEHCDCLQGFQLTHSLG
Rattus_norvegicus    GTMDSVRSGPFGQLFRPDNFIFGQTGAGNNWAKGHYTEGAELVDSVLDVVRKECEHCDCLQGFQLTHSLG
consensus>70         GTMDSVRSGPFGQLFRPDNFIFGQTGAGNNWAKGHYTEGAELVDSVLDVVRKECEHCDCLQGFQLTHSLG

                             150       160       170       180       190       200       210
Homo_sapiens         GGTGSGMGTLLISKIREEYPDRIMNTFSVMPSPKVSDTVVEPYNATLSVHQLVENTDETYCIDNEALYDI
Mus_musculus         GGTGSGMGTLLISKIREEYPDRIMNTFSVMPSPKVSDTVVEPYNATLSVHQLVENTDETYCIDNEALYDI
Rattus_norvegicus    GGTGSGMGTLLISKIREEYPDRIMNTFSVMPSPKVSDTVVEPYNATLSVHQLVENTDETYCIDNEALYDI
consensus>70         GGTGSGMGTLLISKIREEYPDRIMNTFSVMPSPKVSDTVVEPYNATLSVHQLVENTDETYCIDNEALYDI

                             220       230       240       250       260       270       280
Homo_sapiens         CFRTLKLTTPTYGDLNHLVSATMSGVTTSLRFPGQLNADLRKLAVNMVPFPRLHFFMPGFAPLTARGSQQ
Mus_musculus         CFRTLKLTTPTYGDLNHLVSATMSGVTTSLRFPGQLNADLRKLAVNMVPFPRLHFFMPGFAPLTARGSQQ
Rattus_norvegicus    CFRTLKLTTPTYGDLNHLVSATMSGVTTSLRFPGQLNADLRKLAVNMVPFPRLHFFMPGFAPLTARGSQQ
consensus>70         CFRTLKLTTPTYGDLNHLVSATMSGVTTSLRFPGQLNADLRKLAVNMVPFPRLHFFMPGFAPLTARGSQQ

                             290       300       310       320       330       340       350
Homo_sapiens         YRALTVPELTQQMFDAKNMMAACDPRHGRYLTVATVFRGPMSMKEVDEQMLAIQNKNSSYFVEWIPNNVK
Mus_musculus         YRALTVPELTQQMFDAKNMMAACDPRHGRYLTVATVFRGPMSMKEVDEQMLAIQNKNSSYFVEWIPNNVK
Rattus_norvegicus    YRALTVPELTQQMFDAKNMMAACDPRHGRYLTVATVFRGPMSMKEVDEQMLAIQNKNSSYFVEWIPNNVK
consensus>70         YRALTVPELTQQMFDAKNMMAACDPRHGRYLTVATVFRGPMSMKEVDEQMLAIQNKNSSYFVEWIPNNVK

                             360       370       380       390       400       410       420
Homo_sapiens         VAVCDIPPRGLKMASTFIGNSTAIQELFKRISEQFSAMFRRKAFLHWFTGEGMDEMEFTEAESNMNDLVS
Mus_musculus         VAVCDIPPRGLKMASTFIGNSTAIQELFKRISEQFSAMFRRKAFLHWFTGEGMDEMEFTEAESNMNDLVS
Rattus_norvegicus    VAVCDIPPRGLKMASTFIGNSTAIQELFKRISEQFSAMFRRKAFLHWFTGEGMDEMEFTEAESNMNDLVS
consensus>70         VAVCDIPPRGLKMASTFIGNSTAIQELFKRISEQFSAMFRRKAFLHWFTGEGMDEMEFTEAESNMNDLVS

                             430       440
Homo_sapiens         EYQQYQDATVNDGEEAFEDEDEEEINE
Mus_musculus         EYQQYQDATVNDGEEAFEDEDEEEINE
Rattus_norvegicus    EYQQYQDATVNDGEEAFEDEDEEEINE
consensus>70         EYQQYQDATVNDGEEAFEDEDEEEINE
```

Supplemental Figure S4. Conservation analysis of TUBB6 among distinct species. TUBB6 protein sequence is highly conserved among human, mouse and rats.

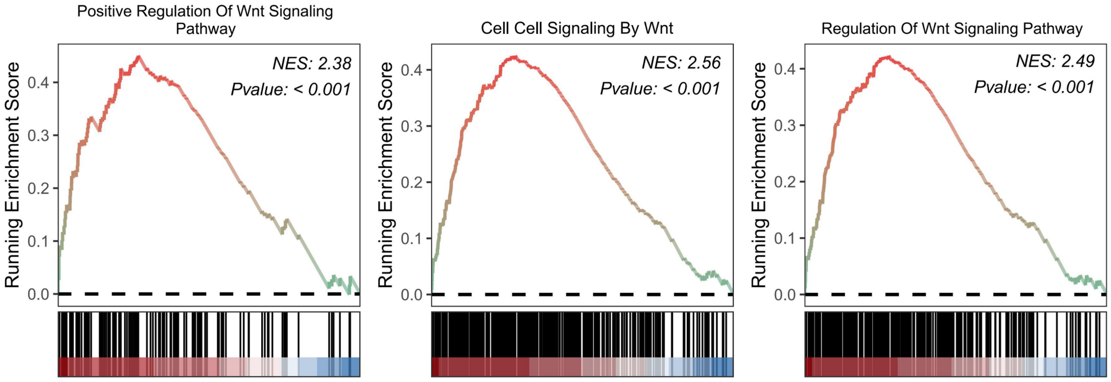

Supplemental Figure S5. GSEA analyses of ECs from PDR patients. GSEA analyses showing activated WNT-related pathways in the CD31-enriched ECs of PDR patients.

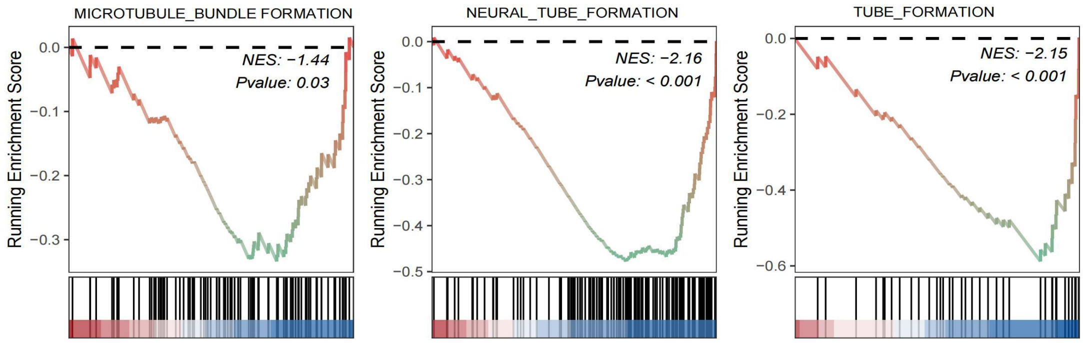

Supplemental Figure S6. GSEA analyses of si-TUBB6-treated ECs. GSEA analyses showing disturbed tubulin-related pathways in HUVECs after TUBB6 knockdown.

**Supplementary Table S1**. Sequences of siRNAs and sgRNA	
Targeted genes	Sequence (5'→3')
In vitro	
scramble siRNA	sense:UUCUCCGAACGUGUCACGUTT
	antisense: ACGUGACACGUUCGGAGAATT
TUBB6-siRNA-1	sense:GUGAGGGCAUGGAUGAAAU
	antisense: CACUCCCGUACCUACUUUA
**TUBB6-siRNA-2**	sense:UGGAGAGAAUCAACGUCUA
	antisense: ACCUCUCUUAGUUGCAGAU
TUBB6-siRNA-3	sense:UGCUGGCCAUCCAGAGUAA
	antisense: ACGACCGGUAGGUCUCAUU
YBX1-siRNA-1	sense:GGACGGCAAUGAAGAAGAUTT
	antisense: AUCUUCUUCAUUGCCGUCCTT
YBX1-siRNA-2	sense:CCACGCAAUUACCAGCAAATT
	antisense: UUUGCUGGUAAUUGCGUGGTT
YBX1-siRNA-3	sense:CUGUGGAGUUUGAUGUUGUTT
	antisense: ACAACAUCAAACUCCACAGTT
**YBX1-siRNA-4**	sense:GUCGACCACAGUAUUCCAATT
	antisense: UUGGAAUACUGUGGUCGACTT
TUBB6-sgRNA	CUGAUCACUUCCCAAAACUU
In vivo	
scramble siRNA	sense:UUCUCCGAACGUGUCACGUTT
	antisense: ACGUGACACGUUCGGAGAATT
**Tubb6-siRNA-1**	sense:CAUUGUCAGUGCACCAGCUTT
	antisense: AGCUGGUGCACUGACAAUGTT
Tubb6-siRNA-2	sense:GGGAACCAGAUCGGUACCATT
	antisense: UGGUACCGAUCUGGUUCCCTT
Tubb6-siRNA-3	sense:UUCGGACAGACGGGUGCCGTT
	antisense: CGGCACCCGUCUGUCCGAATT
Tubb6-siRNA-4	sense:CCGGGGCCCCAUGUCCAUGTT
	antisense: CAUGGACAUGGGGCCCCGGTT

**Supplementary Table S2**. Primers used in this study.		
Gene/RNA	Forward primer (5'→3')	Reverse primer (5'→3')
In vitro		
β-ACTIN	ACCTTCTACAATGAGCTGCG	CCTGGATAGCAACGTACATGG
TUBB6	AAGAGGCCCTAAGAACAGCC	AAACTTGGTGCCGATCTGGT
ESM1	ACAGCAGTGAGTGCAAAAGCA	GCGGTAGCAAGTTTCTCCCC
PLUAR	TGTAAGACCAACGGGGATTGC	AGCCAGTCCGATAGCTCAGG
CXCR4	ACTACACCGAGGAAATGGGCT	CCACAATGCCAGTTAAGAAGA
APLN	GTCTCCTCCATAGATTGGTCTGC	GGAATCATCCAAACTACAGCCAG
FSCN1	CTGCTACTTTGACATCGAGTGG	GGGCGGTTGATGAGCTTCA
TOP2A	ACCATTGCAGCCTGTAAATGA	GGGCGGAGCAAAATATGTTCC
MKI67	GACTTTGGGTGCGACTTGAC	ACAACTCTTCCACTGGGACG
CDK1	CCTATGGAGTTGTGTATAAGGGT	AGCACATCCTGAAGACTGACT
YBX1	GCGGGGACAAGAAGGTCATC	CGAAGGTACTTCCTGGGGTTA
FZD8	ACCCAGCCCCTTTTCCTCCATT	GTCCACCCTCCTCAGCCAAC
WNT3A	CGTGCTGGACAAAGCTACCA	CCAAACTCGATGTCCTCGCT
in vivo		
Gapdh	TGCACCACCAACTGCTTAG	GGATGCAGGGATGATGTTC
Tubb6	CGGCCTGACAACTTCATCTTCG	CCTGAAGACAGTCGCAATGCTC

**Supplementary Table S3**. Antibodies used in this study.				
Anti-protein	Host	Dilution and Application	Supplier	Catalog
In vitro				
GAPDH	Mouse	1:10000, Immunoblotting	Proteintech	60004-1-Ig
TUBB6	Mouse	1:100, Immunofluorescence; 1:2000, Immunoblotting	Proteintech	66362-1-Ig
YBX1	Rabbit	1:100, Immunofluorescence	Proteintech	20339-1-AP
WNT3A	Rabbit	1:5000, Immunoblotting	Proteintech	26744-1-AP
FRIZZLED 8	Mouse	1:5000, Immunoblotting	Proteintech	55093-1-AP
in vivo				
Gapdh	Mouse	1:10000, Immunoblotting	Proteintech	60004-1-Ig
Tubb6	Rabbit	1:200, Immunofluorescence; 1:2000, Immunoblotting	Merck	ZRB2285
Ve-Cadherin	Rat	1:75, Immunofluorescence	Abcam	ab282277
Ng2	Rabbit	1:100, Immunofluorescence	Merck	AB5320
IB4	/	1:200, Immunofluorescence	Vector Laborato ries	FL-1201-.5
